# Impact of BCG vaccination on the repertoire of human γδ T cell receptors

**DOI:** 10.3389/fimmu.2023.1100490

**Published:** 2023-03-28

**Authors:** Mei Xia, Azra Blazevic, Andrew Fiore-Gartland, Daniel F. Hoft

**Affiliations:** ^1^ Department of Internal Medicine, Saint Louis University School of Medicine, Saint Louis, MO, United States; ^2^ Vaccine and Infectious Disease Division, Fred Hutchinson Cancer Research Center, Seattle, WA, United States; ^3^ Department of Molecular Microbiology and Immunology, Saint Louis University School of Medicine, Saint Louis, MO, United States

**Keywords:** γδ TCR, CDR3, BCG, T cell, tuberculosis

## Abstract

**Introduction:**

Tuberculosis (TB) caused by *Mycobacterium tuberculosis* (Mtb) infection is a serious threat to human health. Vaccination with BCG prevents the development of the most severe forms of TB disease in infants and was recently shown to prevent Mtb infection in previously uninfected adolescents. γδ T cells play a major role in host defense at mucosal sites and are known to respond robustly to mycobacterial infection. However, our understanding of the effects of BCG vaccination on γδ T cell responses is incomplete.

**Methods:**

In this study we performed γδ T cell receptor (TCR) repertoire sequencing of samples provided pre- and post-BCG vaccination from 10 individuals to identify specific receptors and TCR clones that are induced by BCG.

**Results:**

Overall, there was no change in the diversity of γTCR or δTCR clonotypes in post- vs pre-BCG samples. Furthermore, the frequencies of TCR variable and joining region genes were minimally modulated by BCG vaccination at either the γTCR or δTCR loci. However, the γTCR and δTCR repertoires of individuals were highly dynamic; a median of ~1% of γTCR and ~6% of δTCR in the repertoire were found to significantly expand or contract in post- vs pre-BCG comparisons (FDR-q < 0.05). While many of the clonotypes whose frequency changed after BCG vaccination were not shared among multiple individuals in the cohort, several shared (i.e., “public”) clonotypes were identified with a consistent increase or decrease in frequency across more than one individual; the degree of sharing of these clonotypes was significantly greater than the minimal sharing that would be expected among γTCR and δTCR repertoires. An *in vitro* analysis of Mtb antigen-reactive γδ T cells identified clonotypes that were similar or identical to the single-chain γTCRs and δTCRs that changed consistently after BCG vaccination; pairings of γTCRs and δTCRs that increased after BCG vaccination were significantly over-represented among the Mtb-reactive γδ T cells (p = 1.2e-6).

**Discussion:**

These findings generate hypotheses about specific γδTCR clonotypes that may expand in response to BCG vaccination and may recognize Mtb antigens. Future studies are required to validate and characterize these clonotypes, with an aim to better understand the role of γδ T cells in Mtb immunity.

## Introduction

Tuberculosis (TB) is one of the top 10 causes of death and the second leading cause from a single infectious agent after COVID-19 (above HIV/AIDS) (1). In 2019, an estimated 10 million people fell ill and more than 1.4 million died from TB worldwide. The total cases included 5.6 million men, 3.2 million women and 1.2 million children.

TB disease is caused by *Mycobacterium tuberculosis* infection (Mtb). The immune responses induced by Mtb during infection, and those that inhibit *in vivo* replication of Mtb, have been studied with the goals of developing novel vaccines, diagnostics, and treatments for drug susceptible and resistant organisms (2–4). Bacille-Calmette Guerin (BCG) is the only vaccine currently licensed for the prevention of TB disease. It is an attenuated mycobacterium that shares many antigens with Mtb and is therefore able to elicit a complex immune response with specific responses against Mtb. Since the TCR repertoire is a mirror of the human T cell response, its characteristics have been widely investigated in infectious and other diseases to study the state of the immune system and the progression of these diseases (5).

The diversity within the TCR repertoire is ensured through somatic recombination of germline‐encoded variable (V), diversity (D), and junctional (J) gene segments. Nucleotide deletions at the coding ends and nucleotide additions at the V(D)J junctions also contribute to TCR repertoire diversity (6). Much of the resulting diversity in the TCR is contained within the third hypervariable complementary determining (CDR3) region, which lies at the intersection between the V, D, J and V, J gene segments within the TCRα/δ and TCRβ/γ chains, respectively. Thus, even when T cell clones express the same V/D/J gene rearrangements, distinct clonotypes can be identified by the unique combinations of their CDR3 sequences (7). The CDR3 region interacts with antigenic peptide/MHC complexes as well as non-peptide antigens; the specificity of the receptor for its cognate antigen depends on the amino acids in the CDR3. Accordingly, the complexity and distribution of TCRs within specific T cell populations will reflect the degree of complexity of the T cell response.

TCR diversity presents unique statistical challenges for analysis. For instance, though deep sequencing provides a large sample size for detecting fluctuations in the frequencies of specific clonotypes over time, individual clonotypes are usually not shared across enough individuals (i.e., limited *publicity*) to allow detection of consistent changes. That means it is possible to detect significant changes in the repertoire of a single individual before and after an immune perturbation, but there is little statistical power to test for significant population-level effects of an intervention, such as BCG vaccination (8).

In the present study, we studied the immune repertoire of γδ T cells from pre- and post-BCG vaccination peripheral blood samples to describe the TCRs of clonotypes that expanded after BCG vaccination. Characteristics of the repertoire including variable and joining segment gene usage, CDR3 sequence diversity, CDR3 length distribution and specific CDR3 sequence abundance did not consistently change after BCG vaccination. However, deep sequencing of individual TCRγ and TCRδ chains identified consistently increased public γ- and δ-chains after BCG vaccination, providing evidence of BCG-induced changes in the immune repertoire of γδ T cells. In addition, paired TCRVγ and TCRVδ chain single cell analyses of mycobacteria-activated γδ T cells confirmed that the most common TCRγ and TCRδ single chains that were significantly and frequently increased after BCG vaccination paired with each other, further indicating that these were relevant for BCG-induced γδ T cell responses.

## Results

### Abundance of γδ T cells responding to Mtb stimulation increased after BCG vaccination

**Figure 1:**
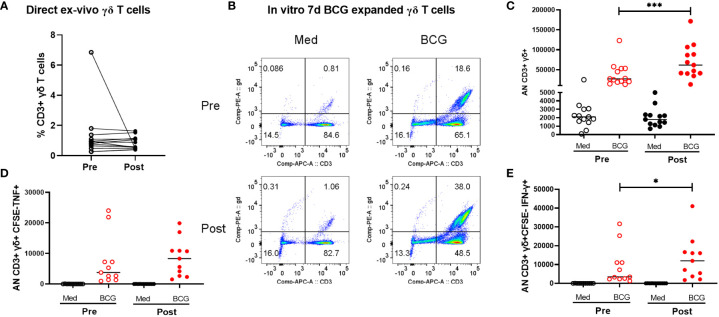
Comparison of peripheral γδ T cells in volunteers pre- and post-BCG vaccination. Direct ex vivo flow cytometric results for 10 volunteers pre- and post-BCG vaccination are shown in panel **(A)** (γδ T cell frequencies of total CD3+ T cells). No significant differences were detected comparing these direct ex vivo samples collected pre- and post-BCG vaccination. However, after BCG stimulation and 7-day expansion periods in vitro as we have previously reported γδ T cell immune responses were significantly increased after BCG vaccination For these in vitro expansion studies, CFSE labeled PBMCs were cultured with BCG (MOI =0.03) for 7 days. PMA (50 ng/mL), ionomycin (1µg/mL) and Gogistop (1,000×) were added to the cells before surface and intracellular staining. Panel **(B)** shows representative flow cytometric results for one - volunteer studied pre- and post--BCG vaccination. Panel **(C)** includes the composite data for all 10 volunteers. Absolute numbers, calculated as the percentages γδ T cells times the total viable cells present on day 7, are shown. Presented in panels **(D, E)** are similar quantifications for the absolute numbers of CFSE- γδ TCR+ cytokine producing- T cells (TNF vs IFN-r producing cells, respectively) from 10 volunteers pre- and post-BCG vaccination. P values were calculated using paired t-test. *p<0.05, ***p<0.001.

All participants in this study were healthy adults who were previously BCG and Mtb-naïve and received a total of two doses of BCG, by intradermal injection, approximately 6 months apart. Demographic data for enrolled volunteers was listed in the **Supplementary Table S8**. QuantiFERON-TB Gold in-Tube (QFT) responses were assessed before and 2 months after both BCG vaccinations (all subjects were negative at both timepoints). Two BCG vaccinations given 6 months apart were chosen for this study to give optimal T cell and antibody responses based on previous experience with BCG vaccination (9–12). Leukapheresis products were obtained prior to the first BCG vaccination and ~2 months after the second BCG vaccination. In this study we focused on the γδ T cells present pre- and post-BCG vaccination. The results shown in **Figure 1** demonstrate that as we have reported before, BCG vaccination led to γδ T cell enhanced expansion capacities after restimulation with BCG for 7 days *in vitro*, indicating BCG vaccine-induced priming of memory, BCG-specific γδ T cells. As shown in **Figure 1A**, no significant differences were noted in the direct ex vivo percentages of CD3^+^ γδ^+^ T cells pre- and post-BCG vaccination without *in vitro* restimulation. However, after *in vivo* BCG vaccination, γδ T cells displayed increased expansion capacity after *in vitro* stimulation with live BCG (**Figures 1B, C**). The percentages of γδ T cells present after 7 days of BCG-induced expansion were more than 20-fold higher. In addition, the absolute numbers of CD3^+^γδTCR^+^CFSE^lo^TNF^+^ and CD3^+^γδTCR^+^CSFE^lo^IFN-γ^+^ T cells present after BCG expansion were increased significantly in post-BCG responders compared with their matched pre-vaccination responses (**Figures 1D, E**). The representative FACS plots were shown in **Figure S8**. Taken together, these results indicated increased BCG-specific memory γδ T cell responses present after BCG vaccination.

### BCG vaccination induced small changes in the proportion of γδ T cells expressing specific variable or joining segment germline genes

To investigate the BCG-induced changes in the γδ T cell response we purified γδ T cells from whole blood and submitted genomic DNA for bulk γ-chain and bulk δ-chain T cell receptor repertoire sequencing. Across the 20 samples (10 individuals pre- and post-BCG) we acquired sequences from a median of 97057 productive γ-chain templates (IQR [75191, 120999], range [13321, 201544]) and 91912 productive δ-chain templates (IQR [69711, 100108], range [18611, 125807]).

**Figure 2:**
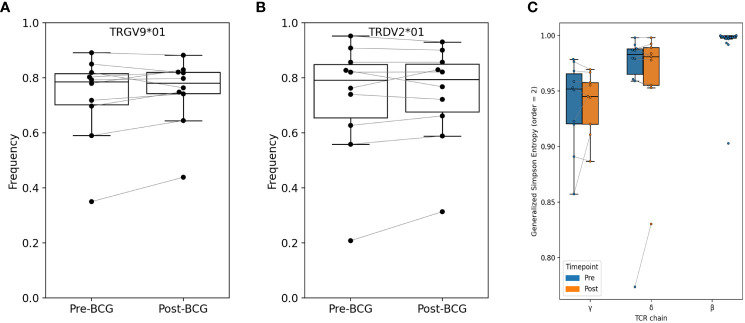
Relative abundance of TRGV9 and TRDV2 genes and diversity among bulk sequenced T cells. Frequency of **(A)** TRGV9 and **(B)** TRDV2 genes are shown before and after BCG vaccination; no significant differences were found (paired signed-rank test, FWER-p > 0.05 and FDR-q > 0.05). Additional V-gene frequency comparisons are provided in **Supplementary Material**. **(C)** Diversity was computed as Generalized Simpson’s Entropy (order = 2, equivalent to Simpson’s Diversity Index); the statistic can be interpreted as the probability of drawing two different TCRs at random from the repertoire. Samples were rarified to 30K productive templates for the purpose of computing diversity. One sample (V005-Post-BCG) was excluded for low sequencing depth. Across participants, repertoire entropy was not significantly altered after BCG vaccination (p > 0.05).

Initially, we compared the observed frequencies of germline variable (V) and joining (J) segment gene usage within the repertoire before and after BCG vaccination (**Figure 2**; **Figures S1, S2**). TCRs using TRGV9 represented the majority of TCRGV clonotypes detected in all subjects with an average of 75% (range 38 - 89%) of the Vγ chains. Similarly, TRDV2, which commonly pair with TRGV9, was the most common Vδ chain which comprised an average of 74% (31 - 93%) of Vδ chains across all individuals. Other common TRGV genes included TRGV4 (1.5 - 7.5%), TRGV3 (1.0 – 6.0%), and TRGV2 (1.5 – 14%); other common TRDV genes included TRDV1 (4.7 – 32%), TRDV8/TRAV38-2 (0.03 – 2.3%), and TRDV5/TRAV29 (0.1 – 0.8%). While many individuals demonstrated a substantial shift in the frequency of TRGV9 TCRs after BCG vaccination (range 0.71 to 1.45-fold change), there were no consistent or significant population-level changes. We noted similar heterogeneity for other TRGV genes, and none showed consistent, significant changes across individuals (beta-binomial [BB] FDRq > 0.2 or paired signed-rank [SR] test, p > 0.05, see *Statistical Methods* for details), with two exceptions of TRGV genes that met statistical criteria despite modest overall changes: TRGV11 (overall 0.85-fold change, BB FDRq = 0.004, SR-p = 0.037) and TRGV10 (overall 1.13-fold change, BB FDRq = 0.079, SR-p = 0.048). Shifts in the frequencies of V and J gene usage in the δ-chain repertoires were also substantial for many individuals (e.g., range 0.67 - 1.73-fold changes in TRDV2 and 0.65 - 1.53-fold changes in TRDV1), however the changes were inconsistent across individuals and not significant in the cohort overall. We also conducted parallel analyses of γ- and δ-chain J gene usage and found no significant population-level changes.

### TCRγ and TCRδ repertoire diversity

We next used several different approaches to consider the hypothesis that specific clonotypes of the γδ TCR repertoire may have increased or decreased in frequency with BCG vaccination. Since clonal expansions can lead to a reduction in overall clonal diversity, we first examined the diversity of TCRγ and TCRδ clonotypes in each sample; here we define a clonotype as a unique amino-acid sequence of either the TCRγ or TCRδ single chain. We used Generalized Simpson’s Entropy (13) as a measure of diversity; order = 2 was used as it represents the probability that two randomly selected TCRs from a repertoire would be different, and is equivalent to the commonly used Simpson’s diversity index. As a comparator we also computed the diversity of TCRβ repertoires from 20 healthy control subjects (14). For diversity comparisons all repertoires were rarified or down-sampled to 30K productive templates, since the number of sequences in a repertoire affects diversity estimates. The greatest diversity was observed among TCRβ repertoires, and diversity among TCRδ repertoires was greater than TCRγ diversity (**Figure 2C**). However, there were no overall significant differences in diversity between pre- and post-BCG repertoires for either TCRγ or TCRδ.

**Figure 3:**
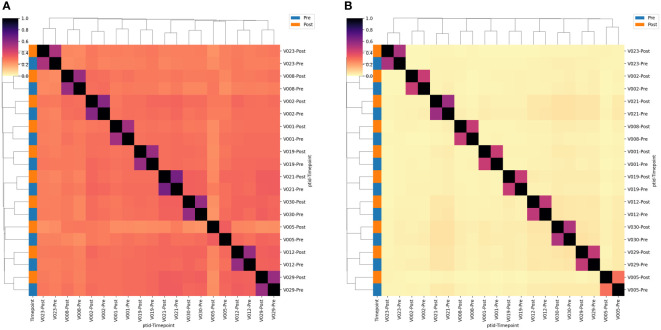
TCR single-chain repertoire sharing. Heatmaps showing the **(A)** TRGV9*01+ and **(B)** TRDV2*01+ clonotypes shared between pairs of samples. Sharing constitutes a single-chain clonotype that matches in the V-gene, J-gene and CDR3 amino acids (proportion shared = 2*N_shared_/[N_A_ + N_B_]). Samples are labeled according to the volunteer number (VX) and the timepoint relative to BCG vaccination. Sample timepoint is also indicated by the color bar.

We next estimated the proportion of clonotypes shared between non-rarefied (i.e., complete) TCR repertoires. For this analysis we split the repertoires into TCRs using TRGV9 vs. non-TRGV9 V-genes and TRDV2 vs. non-TRDV2 V-genes. Sharing of TGRV9 TCRs among the two sample time points from each individual was high, with an estimated median of 70%, however sharing across individuals (i.e., public sharing) was lower with median 30% (**Figure 3**). Sharing was lower among non-TRGV9 clonotypes, both within an individual (30%) and across individuals (10%; **Figure S3**). For TRDV2, sharing was quite high among samples from the same individuals (65%) but was rare across individuals (<5%), suggesting the presence of highly expanded private clonotypes within individuals. Sharing of non-TRDV2 clonotypes was rarely seen between samples, even from the same individual (<2%), though this can partially be attributed to the relatively small fraction of the γδ T cell repertoire that does not use TRDV2 (~20%). As a comparison we also estimated repertoire sharing for all TCRγ and all TCRδ clonotypes from study participants, in addition to TCRβ clonotypes from healthy controls (**Figure S4**); the results were consistent with TCRβ having greater diversity and less public sharing compared to TCRγ and TCRδ. Given the relative paucity of “public” TCRγ and TCRδ clonotypes, the results suggested that it could be difficult to identify unique clonotypes that are shared among individuals and that show a consistent population-level change in frequency after BCG vaccination.

### Changes in clonotype abundance detected in every individual after BCG vaccination

**Figure 4:**
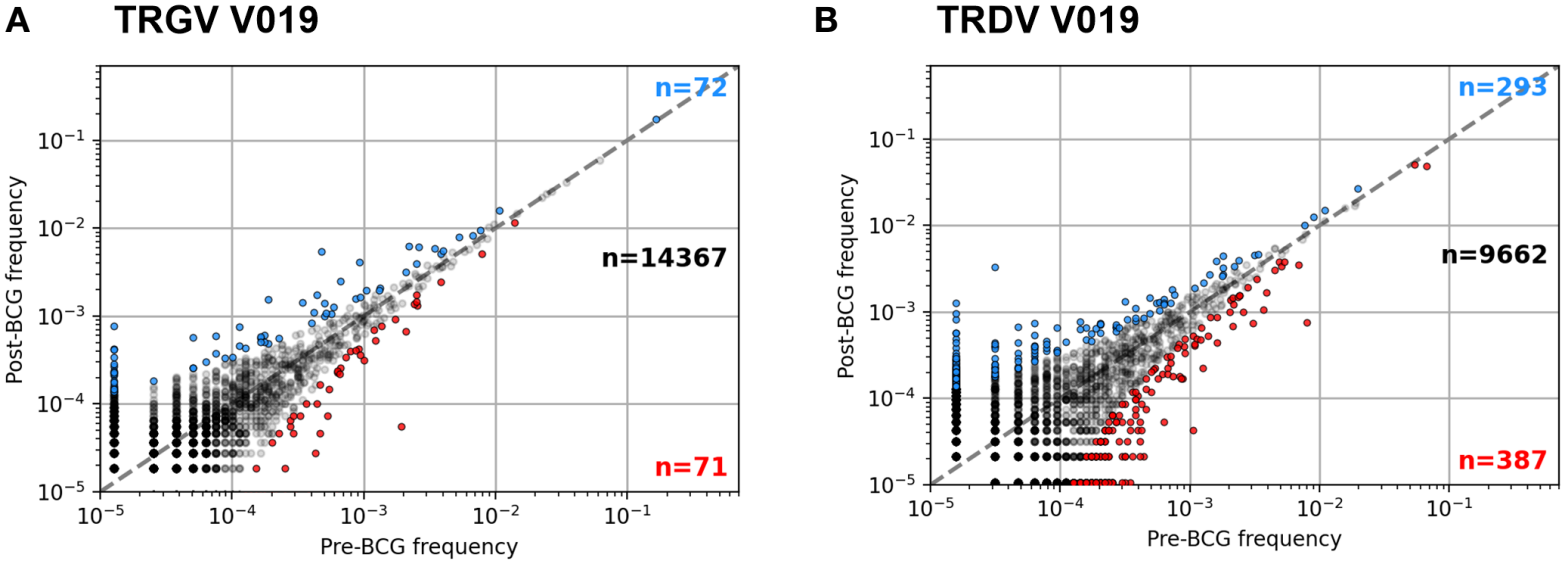
Example frequency scatter plots of per participant clonotype analysis. Pre- and Post-BCG TCRγ **(A)** and TCRδ **(B)** repertoires were analyzed from each participant. Figure shows example frequency scatter plots for one participant (V019); analysis of additional participants provided in the **Supplementary Materials**. Each symbol represents one TCR single chain clonotype plotted as pre vs. post BCG frequency. Symbols colored blue (red) show clonotypes with an FDR-adjusted q-value < 0.05 indicating a significant increase (decrease) in frequency after BCG vaccination (Fisher’s exact test). For plotting, a pseudocount of one was added to the observed counts of each TCR; as a result, the data at the lowest frequency represents TCRs that were not observed in the respective sample (pre- or post-BCG).

We next sought to identify unique clonotypes whose abundance changed after BCG vaccination. An analysis of each participant compared the frequency of each clonotype in the samples collected pre- and post-BCG vaccination; a positive odds-ratio indicated an increase in abundance post-BCG (example **Figure 4**; all analyses **Figure S5**). Participants had a median of 15,474 unique TCRγ sequences (IQR 10,948 – 18,507) and 11,062 unique TCRδ sequences (IQR 6,584 – 13,462). A median of 0.59% of TCRγ sequences (74 TCRs) were significantly increased and 0.47% decreased (60 TCRs) in the post-BCG samples (Fisher’s exact test, FDR-adjusted q < 0.05). Results were similar for TCRδ with medians of 3.0% (285) of sequences significantly increased post-BCG and 3.9% (346) decreased. It is important to note that these changes cannot necessarily be attributed to the vaccination; the longitudinal dynamics of specific γδ TCR clonotypes has not been well described and changes could be attributable to other factors, including random fluctuation.

**Figure 5:**
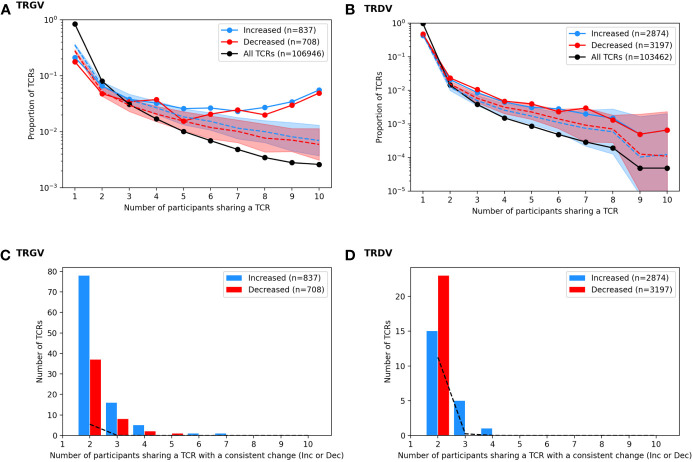
Analysis of TCRs with a significantly different frequency at the pre vs. post BCG time points. TCRγ and TCRδ repertoires were analyzed to identify TCRs from each participant that either increased or decreased significantly after BCG vaccination (Fisher’s exact test, FDR-adj Q-value < 0.05). **(A)** A histogram shows the proportion of all unique TCRγ chains (black) that were observed in one individual or shared across multiple individuals (x-axis, sharing number). A histogram was also created for the set of TCRs that increased after BCG vaccination (blue, n = 837) and the set of TCRs that decreased after BCG vaccination (red, n = 708). Proportions are represented on a log-scale y-axis. A simulation under the null-hypothesis (i.e., random TCRs were detected as significantly increased or decreased) was repeated 1000 times, with a histogram computed for each iteration; the median proportion is plotted for the randomly simulated increased (blue dashed line) and decreased (red dashed line) TCRs with a shaded region indicating the 97.5th and 2.5th quantiles. **(B)** For each TCR that increased significantly in more than one individual, the number of participants that also showed an increase (blue) in that TCR was tallied; the same analysis was conducted for decreased TCRs (red) yielding a count histogram of the consistency with which each TCR changed after BCG vaccination. Consistency was also quantified from the null simulated data (black dashed line). **(C, D)** A parallel set of analyses were conducted based on the TCR-delta chain repertoires.

One way to link a specific clonotype to BCG vaccination would be to observe a consistent increase after vaccination in multiple individuals. This would unlikely due chance because the majority of TCRγ and TCRδ chains are not shared by more than one individual and a small minority are shared by two or more individuals (**Figure 5** and **Table 1**): e.g., among TCRγ and TCRδ chains, <2% and <1%, respectively, were observed among more than two participants. However, we noted that many of the TCRγ and TCRδ chains that significantly increased or decreased post-BCG were among these rare “public” TCRs: 14% of TCRγ and 1.1% of TCRδ chains were significantly changed in frequency after BCG vaccination in more than one individual. To understand the significance of this observation, we conducted a simulation study under the null hypothesis that random TCRs from each individual were identified as significantly changed by randomly re-assigning the TCR odds-ratios among the unique TCRs of each individual. While identifying significant TCR sequences at random from each repertoire was slightly biased towards shared TCRs (**Figure 5**, dashed lines averaging 100 repetitions), the actual TCRs with a significant change were notably more public. We also observed that the CDR3 region of TCRs with a significant change after BCG vaccination were slightly shifted in length from the distribution of lengths overall (**Figure S6**); the significantly changed TCRγ CDR3s were ~1 aa longer while the TCRδ CDR3s were ~1 aa shorter, and the difference was reflected both in TCRs that increased and decreased significantly after BCG vaccination. TCRγ and TCRδ clonotypes with significant and consistent (≥3 individuals) changes after BCG vaccination were listed in **Table 1**. Together these analyses suggested that there was something distinct about the TCRs that were significantly changed in abundance post-BCG.

**Table 1:** TCRγ and TCRδ clonotypes with significant (FDR-q < 0.05) and consistent (≥3 individuals) changes after BCG vaccination.

	V-gene	J-gene	CDR3 AA	Publicity	Consistency	Change Post-BCG
**TCRγ**	TRGV9*01	TRGJP*01	CALWEAELGKKIKVF	10	7	Increasing
	TRGV9*01	TRGJP*01	CALWEVAQELGKKIKVF	10	3	Decreasing
	TRGV9*01	TRGJP*01	CALWEVWELGKKIKVF	10	3	Decreasing
	TRGV9*01	TRGJP*01	CALWESQELGKKIKVF	10	4	Increasing
	TRGV9*01	TRGJP*01	CALWEVAELGKKIKVF	9	3	Increasing
	TRGV9*01	TRGJP*01	CALWEVTELGKKIKVF	10	4	Increasing
	TRGV9*01	TRGJP*01	CALWEVQQELGKKIKVF	10	3	Decreasing
	TRGV9*01	TRGJP*01	CALWEVSELGKKIKVF	10	3	Increasing
	TRGV9*01	TRGJP*01	CALWEVVELGKKIKVF	10	3	Decreasing
	TRGV9*01	TRGJP*01	CALWGLQELGKKIKVF	7	3	Increasing
**TCRδ**	TRDV2*01	TRDJ1*01	CACDTLLGDTDKLIF	8	3	Increasing
	TRDV2*01	TRDJ1*01	CACDTVGDTDKLIF	9	3	Increasing
	TRDV2*01	TRDJ1*01	CACDPLLGDTDKLIF	9	3	Increasing
	TRDV2*01	TRDJ1*01	CACDTLGDTDKLIF	10	4	Increasing
	TRDV2*01	TRDJ1*01	CACDTLGDTRDKLIF	6	3	Increasing

**Figure 6:**
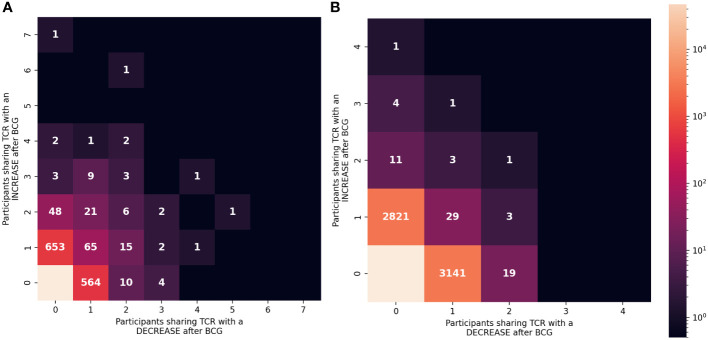
Consistency of the changes observed after BCG vaccination among TCRγ and TCRδ repertoires. **(A)** TCRγ and **(B)** TCRδ repertoires were analyzed to identify TCRs from each participant that either increased or decreased significantly after BCG vaccination (Fisher’s exact test, FDR-adj q-value < 0.05). Each unique TCR was categorized based on the number of individuals that shared the TCR and showed a significant increase or decrease after BCG vaccination. Each category (one box) is colored based on the number of unique TCRs (out of 106,946 TCRγ or 103,462 TCRδ clonotypes) with the corresponding number of people with an increase (y-axis) or decrease (x-axis). Leftmost column shows TCRs that exclusively increased in ≥1 participant and bottom row shows TCRs that exclusively decreased in ≥1 participant (except bottom left box which indicates that the majority of TCRs did not significantly change in frequency after BCG vaccination.

To focus on TCRs with a potential direct link to BCG vaccination we categorized sequences based on how often they were detected as significantly increased or decreased among the 10 participants (**Figure 6**). Of the 837 TCRγ sequences that were significantly increased in the whole cohort, there were 101 (12%) TCRs that were significantly increased in more than one individual. Similarly, among the 708 TCRγ sequences that were decreased across the cohort, 48 (6.7%) were consistently decreased in more than one individual. We refer to these as TCRs with a “consistent” change. Using the simulation of the null hypothesis described above we noted that it was rare that identical sequences were selected from multiple individuals for a consistent change after BCG vaccination; across 100 repetitions there were on average only 10 (1%) TCRs consistently increased in more than one participant, and none were seen in 3 or more participants. By this measure, the consistency observed for a subset of the TCRs that changed after BCG vaccination was unlikely to be explained by chance alone.

For TCRδ, of the 2874 TCRs that were significantly increased, 21 (0.7%) were increased in >1 participant; among the 3197 decreased sequences 56 (1.8%) were consistent in >1 participant. While these were not greater than what was expected by chance alone, there were 6 TCRδ sequences that were consistently increased in 3 or more individuals and zero sequences that were consistently decreased; in the repeated simulations there were no sequences observed to consistently change in 3 or more participants. These analyses further support that the TCRs with a consistent change after BCG (≥2 individuals) may be related to the vaccination.

### Pairing of TCRγ and TCRδ chains with consistent increases after BCG vaccination

To further investigate the biological significance of the TCRγ and TCRδ sequences that changed after BCG vaccination, we focused on sequences that were either increased or decreased in ≥2 individuals, and that were not significantly changed in the opposite direction in any other individual (TCRγ: 54 increased, 14 decreased; TCRδ: 16 increased, 19 decreased; **Figure 6**). All the sequences were derived from γ_9_δ_2_ T cells, which also contributed the majority of all TCRs that were significantly changed post-BCG. We hypothesized that if these single-chain TCRγ and TCRδ sequences were from a truly public γδ T cell clonotype, we would be able to find pairings of these single chains in single-cell data. We conducted exploratory analyses with four individuals to identify and sequence the TCRs from γδ T cells that were activated by stimulation with mycobacterial antigens. From each individual, pre- and post-BCG whole PBMCs or γδ T cells purified by negative selection were stimulated with either whole Mtb lysate (whole PBMC) or purified autologous monocytes infected with Mtb or pulsed with different mycobacterial antigens (purified γδ T cells). We sequenced 2708 cells with complete TCRγ and TCRδ sequences, with a median of 644 per individual. There were insufficient numbers of cells to make strong claims about the specificity of the stimulations, however, we were able to use the paired-chain data to identify real pairings of the single-chains we observed in the bulk repertoires.

To match pairs of TCRγ and TCRδ sequences present within the single-cell data, we made potential pairings of all the TCRγ and TCRδ sequences identified in the single chain data that were consistently increased or decreased post-BCG (68 TCRγ x 35 TCRδ = 2380 potential pairings). Of these potential pairings, 143 were almost identical to a real pairing observed in the single-cell datasets (≤12 TCRdist units ≈ ≤1 amino-acid substitution across both chains; **Figure S7**). 51% (73) of the observed real pairings were between TCRγ and TCRδ chains that had both increased after BCG vaccination. In contrast, only 8% (12) of the real pairings were between chains that both decreased after BCG vaccination, and only 41% (58) had one chain with a consistent increase and one with a consistent decrease. Overall, there were significantly more paired TCRs with a γTCR and δTCR that had both demonstrated a consistent increase after BCG vaccination; under a null-hypothesis of randomly observed pairings only 36% would be expected to have a TCRγ and TCRδ with consistent increases after BCG vaccination compared to the 51% observed (χ^2^ test, p = 1.2e-6).

There were two potential γδTCR pairings that shared identical amino-acid similarity with observed single-cell sequences (**Table 2**). One clonotype had the TCRγ CDR3 sequence **CALWEAELGKKIKVF** which was observed in all 10 individuals, and which increased in 7 of 10 of them. This TCRγ sequence was found to pair with a TCRδ sequence **CACDTVGVTDKLIF**, which increased in 2 out of the 6 individuals in which it was observed. The second potential pairing with an exact clonotypic match in the observational data was also between public TCRγ (**CALWEVQAELGKKIKVF**) and TCRδ (**CACDTLGDTDKLIF**) chains that both increased consistently.

**Table 2:** Paired TCRγ and TCRδ single-chains observed in single-cell, paired-chain data.

TCRγ			TCRδ			
BIOID	Consistency	Change Post-BCG	BIOID	Consistency	Change Post BCG	Minimum distance*
TRGV9*01|TRGJP*01|CALWEAELGKKIKVF	7	Increasing	TRDV2*01|TRDJ1*01|CACDTVGVTDKLIF	2	Increasing	0
TRGV9*01|TRGJP*01|CALWEVQAELGKKIKVF	2	Increasing	TRDV2*01|TRDJ1*01|CACDTLGDTDKLIF	4	Increasing	0

*Minimum distance of zero indicates that the exact paired-chain clonotype (amino-acids) was observed in the single-cell data.

## Discussion

γδ T cells are attractive candidates for novel vaccines against TB because they are not MHC I restricted, and thus, can be broadly stimulated in the target population (15). Early studies identified simple phosphoantigens such as isopentenyl pyrophosphate (IPP) as potent polyclonal stimulators of virtually all γ_9_δ_2_ T cells (16). However, our previous studies have shown that only a subset of the IPP or other phosphoantigen-responsive γδ T cells expressing a more limited diversity of γδ TCR, are capable of recognizing Mtb-infected human monocytes and mediating inhibitory effects on intracellular mycobacteria (17). In this earlier work we established a reverse genetics strategy cloning the δ_2_ and γ_9_ CDR3 PCR fragments for sequencing from selected pairs of BCG- and IPP-stimulated long-term γ_9_δ_2_ T cell lines generated from PPD+ volunteers. We found that the CDR3 sequences of BCG-expanded populations were more oligoclonal while the CDR3 sequences of IPP-expanded populations were more polyclonal. However, due to the limitations of the throughput of this method, and the lack of parallel studies done with samples obtained prior to mycobacterial sensitization from the same individuals, we obtained limited information about *in vivo* γδ TCR repertoires and how they are altered by BCG vaccination or Mtb infection. In the current study we utilized a much more comprehensive strategy to investigate *in vivo* γδ TCR repertoires and how they change after BCG vaccination. We found little evidence that these changes induced by BCG are reflected in the overall balance of γδ T cells in the blood. However, we do show that BCG vaccination may induce a private repertoire of γ_9_δ_2_ T cells that change in frequency following vaccination.

With the development of sequencing technology, deep sequencing has already been widely applied in the analysis of TCR repertoire and immunoglobulin repertoire. However, most previous investigations using deep sequencing of TCR repertoires have focused on αβ T cell repertoires. Papadopoulou (18) found that BCG vaccination did not affect the expansion of public fetal γ_9_δ_2_ T cells and their functional differentiation by comparing 10-wk-old infants from South Africa with or without BCG vaccination at birth intradermally. The absence of clonotypic expansions after infant BCG vaccination could be due to a lack of depth of analysis necessary to identify the relevant clonotypes, lack of full maturation of γδ T cells in infants with poor expansion capacity after BCG vaccination, and/or the relative insufficiency of the γ_9_δ_2_ T cell subset present in infancy compared with other subsets of γδ T cells. Seshadri (19) et al. used peripheral blood mononuclear cells (PBMC) derived from a Phase I study of South African adults in which samples were archived at baseline, 3 weeks, and 52 weeks post-BCG revaccination. They found TRDV*02+CACDTLLGDTRTDKLIF to be a meta-clonotype associated with BCG vaccination, enriched post-BCG in 9 of 17 samples and persisting for 1 year. These results strengthen our conclusions in the present manuscript that the Vγ_9_ and Vδ_2_ chains that were consistently increased in our current study may be public and relevant for the BCG/TB responses in highly diverse populations.

Perhaps γδ TCR repertoire studies of human lung airway cells would be an optimal focus for identifying specific clonotypes involved in pulmonary immunity against Mtb. However, bronchoalveolar (BAL) studies were not applied in our current study because among the 40-50 BALs we have done on BCG vaccinated persons over the past 10 years, γδ T cells were rarely detected in the BAL cell samples. Silver (20–23) has done BAL samples in persons with LTBI and not seen many γδ T cells in BAL. However, an older report (24) studied both blood and BAL and found a striking absence of γδ T cells in both locations in patients with active TB disease, suggesting that the absence of γδ T cells during response to disease was associated with TB susceptibility. Also, it is possible that protective γδ T cells could be present in the lung parenchyma after BCG vaccination, and not in the alveolar spaces amenable to BAL sampling. In addition, Behar et al. have recently demonstrated that the lungs of HIV-negative individuals have a predominance of Vδ1 (and Vδ3) usage, a preference for Jδ1, and clonal expansions, while blood typically contains predominantly γ9δ2 T cells (25). In any case, more sensitive methods for studies of γδ T cell repertoire studies in human airways are needed to address this important question of whether specific γδ T cells are capable of providing lung mucosal immunity.

In the current study, we performed deep γδ TCR repertoire sequencing of blood samples obtained pre- and post- BCG vaccination from 10 adult individuals recruited to the Saint Louis University Center for Vaccine Development to identify specific receptors and TCR clones that are induced by BCG. We found that the γδ TCR repertoire was quite dynamic, with hundreds of receptors increasing or decreasing in frequency after BCG vaccination. Though many of these dynamic TCRs were private and could not be linked directly to BCG vaccination, there was a number of public TCRγ and TCRδ with changes after BCG vaccination that were consistent across the individuals in the study. It was also notable that a higher proportion of the consistent changes were increases after BCG vaccination, consistent with the process of antigen-specific expansion. Though we had hypothesized that there would be TCRs with an increase in frequency after BCG vaccination, we had not expected to observe so many TCRs with a decrease in frequency after BCG vaccination. These decreases could partially be explained by the statistics of a compositional analysis, which requires that increases in one population are accompanied by decreases in every other population. It is also possible that many of the decreases and increases were the result of stochastic fluctuations in the repertoire, unrelated to BCG vaccination; that would also explain why many of the changes were private or inconsistent across individuals. Additionally, it is important to remember that the changes in frequency after BCG were associated with unpaired TCRγ or TCRδ sequences, meaning that the changes we observed in bulk sequenced single-chain data may reflect fluctuations of many different γδ T cell clonotypes in the repertoire.

Engineered T cell receptors have tremendous therapeutic potential for targeted, T cell-mediated killing of infected cells (26). Therefore, one of our goals was to try to identify γδ TCRs relevant for TB-protective immunity. Though we were not able to generate paired-chain TCR sequences from all participants, with limited single-cell paired-chain analysis we were able to identify two pairs of TCRγ and TCRδ sequences expressed by individual γδ T cells, that were responsive to TB-related stimuli and that were found among the public BCG-increased single γ and δ chain bulk repertoire analyses. Although our results suggest that these clonotypes were expanded by BCG vaccination, it will be important to validate these conclusions and define the antigen reactivity of these clonotypes.

To our knowledge, our study is the first to evaluate the effects of BCG on T cell responses in humans from the US. Therefore, detection of these specific γδ chains in different populations suggests broad relevance of our findings. Also, other findings (27) focused on persons exposed to Mtb while the current work has focused on BCG vaccination. Identification of paired γδ chains associated with the responses to both Mtb infection and BCG vaccination further indicate the broad relevance of our findings.

Furthermore, we are the first to sequence single-cell TCR γ and δ chains using multiplex PCR strategies associated with Sanger sequencing or high-throughput sequencing. Hans and colleagues have implemented a PCR-based single cell barcoding strategy to pool all the amplicons and sequence them by NGS (28). A barcode is a short nucleotide sequence that uniquely tags cell transcripts and is used to set up high-throughput methodologies to identify clones sharing the same α and β TCR sequences and applied to tumor-infiltrating lymphocytes from breast and lung cancer. We present an integrated analytical strategy that effectively resolves both the low sensitivity of single-cell sequencing data and the reduced specificity of single-chain sequencing. We show that this integrated dataset enhances the sensitivity and accuracy of sequence detection and identification of the TCR clonotypes. We propose that our approach provides a new tool by bridging the gap between old (bulk) and new (single-cell) methodologies for TCR clonotype identification studies.

The present study has several important limitations. First, the sample size was not sufficient for definitively identifying public TCR sequences with significant changes after BCG vaccination. Ideally, we might have focused primarily on population-level analyses of public TCRs. However, due to the incredible diversity of TCR sequences and the sample size, it was not possible to identify with certainty, which TCRs were increased after BCG vaccination, accounting for multiple hypothesis testing across the public repertoire. Instead, we leveraged the paired pre- vs. post-BCG samples from each individual to identify candidate TCRs that could then subsequently be evaluated in the population. Though this strategy yielded promising results, it requires validation in additional cohorts.

Secondly, though we were able to use single-cell sequencing to identify two candidate γδ TCR clonotypes that may have expanded consistently after BCG vaccination, the application of the method to a small number of individuals and the limited number of cells that were sequenced made it challenging to identify γδ T cells for many of the γTCRs or δTCRs which increased after BCG vaccination. However, this small dataset shows that γδ T cells can be stimulated and sequenced to identify paired-chain TCR sequences whose specificity could subsequently be evaluated experimentally. In future, investigations with much larger sample sizes of subjects, and deeper sampling of the repertoire must be studied to achieve the potential of this powerful technology.

Overall, we provide additional direct evidence that γδ T cells are expanded by BCG vaccination and specifically that clonotypes expressing public Vγ_9_ and Vδ_2_ TCRs may be relevant for protective immune responses induced by BCG or Mtb infection in highly diverse populations. Two potential γδ TCR pairings that shared identical amino-acid similarity with observed single-cell sequences were identified. These paired TCR sequences can be used to generate γδ T cells with an engineered TCR for molecular definition of their antigen and presentation requirements. In addition, these engineered γδ T cells could be useful as therapeutics for use in patients with drug-resistant TB and TB-HIV co-infections.

## Materials and methods

### Clinical cohort

Healthy adult volunteers were recruited according to protocols approved by the Saint Louis University Institutional Review Board #26646 and #26645. Written consent from the volunteers was obtained according to the principles expressed in the Declaration of Helsinki. Ficoll-Paque (GE Healthcare, Piscataway, NJ) was used to obtain PBMC from leukapheresis samples (29).

### Reagents

Connaught BCG was used for *in vitro* expansion of mycobacterium-specific T cells. The following antibodies from BD Bioscience were used for flow cytometric analyses: anti-γδ T cell receptor (TCR) antibody-phycoerythrin (PE) (clone 11F2), anti-γδ TCR APC (Clone B1), anti-αβ TCR antibody-fluorescein isothiocyanate (FITC) (clone B3), anti-CD3 antibody-peridinin chlorophyll protein (PerCP) (clone SK7), anti-CD4 Pacific Blue (clone RPA-T4), anti-CD8 antibody–PE-Cy7 (clone RPA-T8), anti-IFN-γ APC antibody-Alexa Fluor 700 (clone B27), and anti-TNF antibody-FITC(clone Mab11) Carboxyfluorescein succinimidyl ester (CFSE) was obtained from Molecular Probes (Eugene, OR). Phorbol myristate acetate (PMA; Sigma-Aldrich), ionomycin (Sigma-Aldrich), and the Cytofix/Cytoperm kit (BD Biosciences) were used in the preparation of cells for intracellular staining.

### CFSE-based flow cytometric assay to study the Mtb-specific T cells

We followed methods previously described for a CFSE-based T-cell proliferation assay (30) to measure T cell proliferation to live BCG. Briefly, PBMC were labeled with CFSE (Molecular Probes) as recommended by the manufacturer. CFSE-labeled PBMC (1 × 106/ml) were stimulated with live BCG with MOI 0.03 for 7 days at 37°C. On day 7, the cells were restimulated with PMA (50 ng/ml) and ionomycin (750 ng/ml) in the presence of Golgi Stop (0.7 μl/ml) for additional 2 h and studied for intracellular IFN-γ or TNF expression. Flow cytometric acquisition was performed on a multicolor BD FACS Canto II instrument, and analyses were done using FlowJo (Tree Star) software. A minimum of 10,000 events were acquired. Lymphocyte population was identified based on forward and side scatter. Then, CD3^+^ γδTCR^+^ T cells were regated, and then the CFSE low (CFSE^lo^) proliferating populations positive for IFN-γ and/or TNF were identified as effector subsets. The absolute numbers of effector populations were calculated by multiplying the percentage of each subset obtained with flow cytometry by the trypan blue-determined total viable cell counts.

### γδ T cell isolation, genomic DNA extraction

γδ T cells were positively selected as previously described (Arruda, Gaballa, & Uhlin, 2019) using the Anti-TCR γδ MicroBead Kit (130-050-701, Miltenyi Biotec) and LS columns (130-050-701, Miltenyi Biotec) according to the manufacturer. Purity was confirmed and gDNA was extracted using the QIAamp DNA Blood Mini Kit (Qiagen Cat# 51104) and stored at−20°C. NanoDrop 2000 (Thermo Fisher Scientific) was used to determine DNA concentration and purity.

### Adaptive immunoSEQ γ and α/δ bulk TCR sequencing

TRG and TRD CDR3 regions were amplified and sequenced from gDNA by Adaptive Biotechnologies using the immunoSEQ platform at survey resolution. The same amount of gDNA was used for all samples. Briefly, multiplexed V and J gene primers were used to allow deep, quantitative, and nonbiased amplification of the γ and δ chain sequences for high-throughput sequencing.

#### γδ T cell stimulation

In some experiments PBMCs were stimulated with different antigens 50pM HMBPP, 1ug/ml mGLP or 20ug/ml Mtb WL for 7days, then stimulated cells were harvested and stained for 20 mins at room temperature for flow cytometric analysis and cell sorting. In other experiments γδ T cells were enriched by MACS using TCRγ/δ+ T-cell isolation kit (130-092-892, Miltenyi Biotec) and placed on LS columns to separate γδ T cells in the unlabeled fraction from other cells attached to magnet. The lower concentration of adherent monocytes (~1.5 × 104/well in 96-good plates) was prepared as previously described (9). Monocytes were infected overnight with Mtb at a multiplicity of infection (MOI) of 3 or pulsed with different antigens 500pM HMBPP, 1ug/ml mGLP or 20ug/ml Mtb WL. Extracellular bacilli or extra antigens were washed away, and then infected cells or pulsed cells were cocultured with enriched γδ T cell at 37°C with 5% CO2 for 18h. Then stimulated γδ cells were harvested and stained for 20 min at room temperature for flow cytometric analysis and cell sorting.

#### Single-cell sorting

Stimulated PBMCs or γδ cells were stained with the following antibodies: Live-dead, (L34966, Invitrogen), CD3(clone UCHT1), CD19-PerCP (clone HIB19), γδ TCR-PE (clone 5A6.E9), αβ TCR-PE-Cy7(clone IP26), CD69-APC (clone FN50), CD137-BV650 (clone4B4-1). After staining, cells were washed twice and stored on ice until acquisition and sorting on a FACS Aria Fusion cell sorter (BD Biosciences). CD3^+^CD19^-^αβ^-^γδ^+^CD69^+^CD137^+^were single cells sorted directly into individual wells in a 96-well plate (Eppendorf 951020303) containing RT-PCR buffer.

#### Barcode-enabled high throughput single-cell TCR determination

Single T cells are sorted into 96-well PCR plates and sequencing is performed as described (28, 31), except human γδ TCR specific primers are used for this study. γδ TCR primers and the sequencing reaction protocols are offered by Dr Chien from Stanford University. Briefly, for the first reaction, reverse transcription and preamplification were performed with a One-Step RT-PCR kit (Qiagen) using multiplex PCR with multiple Vγ and Vδ region primers, Cγ and Cδ region primers. A 1-μl aliquot of the first reaction product is then used in a second PCR reaction, with nested primers for TCR genes. A third reaction is then performed that incorporates individual barcodes into each well. The products are combined, purified, and sequenced using the Illumina MiSeq platform. The resulting paired-end sequencing reads are assembled and de-convoluted using barcode identifiers at both ends of each sequence by a custom software pipeline to separate reads from every well in every plate. The resulting sequences are analyzed using VDJFasta (32), which we have adapted to resolve barcodes and analyze sequences with a customized gene-segment database. The CDR3 nucleotide sequences are then extracted and translated.

#### γδ TCR sequence analysis

Analysis of bulk γ- and δ-chain TCR sequencing data was conducted based on the usage of specific germline V and J gene usage, as well as their specific CDR3 amino-acid sequences. Changes in gene usage in the repertoire pre vs. post BCG were tested using a Wilcoxon signed-rank test with p-values from the models subject to multiplicity adjustment to control the false-discovery rate (FDR). Fisher’s exact test was used to test for changes in an individual’s pre vs. post TCR repertoire (also subject to multiplicity adjustment). A TCR similarity metric [TCRdist (8)] was used to identify paired-chain single-cells with TCRs similar to those single-chains that were consistently increased or decreased; a radius of <12 TCRdist units (~1 aa substitution in the CDR3) was used to signify similarity. For generation of graphs and statistical analysis we used Python/matplotlib and GraphPad Prism version 9.0.0 for Windows, GraphPad Software, San Diego, California USA, www.graphpad.com.

## Data availability statement

The original contributions presented in the study are publicly available. This data can be found here: https://doi.org/10.6084/m9.figshare.22046690.

## Ethics statement

The studies involving human participants were reviewed and approved by Saint Louis university Institutional Review Board. The patients/participants provided their written informed consent to participate in this study.

## Author contributions

AF-G and DH designed key experiments. AB and MX collected the human leukapheresis samples and performed the flow assay. MX performed bulked and barcode-enabled high throughput single-cell TCR determination experiments. AF-G run γδ TCR sequence analysis. All authors reviewed draft before submission. All authors contributed to the article and approved the submitted version.
