# Supplementary material for: Impact of BCG vaccination on the repertoire of human γδ T cell receptors
Source: Front Immunol. 2023 Mar 28;14:1100490. doi: 10.3389/fimmu.2023.1100490 (PMC10089282; doi:10.3389/fimmu.2023.1100490)
Supplement: Supplementary file 1 [file DataSheet_1.docx]

**SUPPLEMENTARY MATERIAL**

**Impact of BCG vaccination on the repertoire of γδ T cell receptors**

Running title: Association of γ- and δ-chain TCR sequences with BCG vaccination

Mei Xia^1^, YH Chien^3,^ Andrew Fiore-Gartland^4 *†^, Daniel F. Hoft^1,2*†^

^1^Department of Molecular Microbiology and Immunology, Saint Louis University School of Medicine, Saint Louis, MO. USA

^2^Department of Internal Medicine, Saint Louis University School of Medicine, Saint Louis, MO. USA

^3^ Program in Immunology, Department of Microbiology and Immunology, Stanford University, Stanford, CA.USA

^4^ Vaccine and Infectious Disease Division, Fred Hutchinson Cancer Research Center, Seattle, WA. USA.

*†Co-correspondence to: Dr. Daniel F. Hoft ([Daniel.Hoft@health.slu.edu](mailto:Daniel.Hoft@health.slu.edu)) And Dr. Andrew Fiore-Gartland ([agartlan@fredhutch.org](mailto:agartlan@fredhutch.org))

Key words: γδ TCR, CDR3, BCG, tcrdist

# SUPPLEMENTAL TABLES

Supplemental Table S1. Number of productive templates for TCRγ and TCRδ sequencing

Supplemental Table S2. TCRγ clonotypes (by individual) with a significant change in pre vs. post-BCG relative abundance (FDR-adjusted q<0.05)

Supplemental Table S3. TCRδ clonotypes (by individual) with a significant change in pre vs. post-BCG relative abundance (FDR-adjusted q<0.05)

Supplemental Table S4. Consistently increased or decreased TCRγ clonotypes in pre vs. post-BCG samples

Supplemental Table S5. Consistently increased or decreased TCRδ clonotypes in pre vs. post-BCG samples

Supplemental Table S6. Potential pairings of TCRγ and TCRδ chains observed in limited paired-chain sequencing.

Supplementary Table S7. Comparison of TCRδ results with James et al.

Supplementary Table S8. Demographic data for enrolled volunteers.

# SUPPLEMENTAL FIGURES

| A 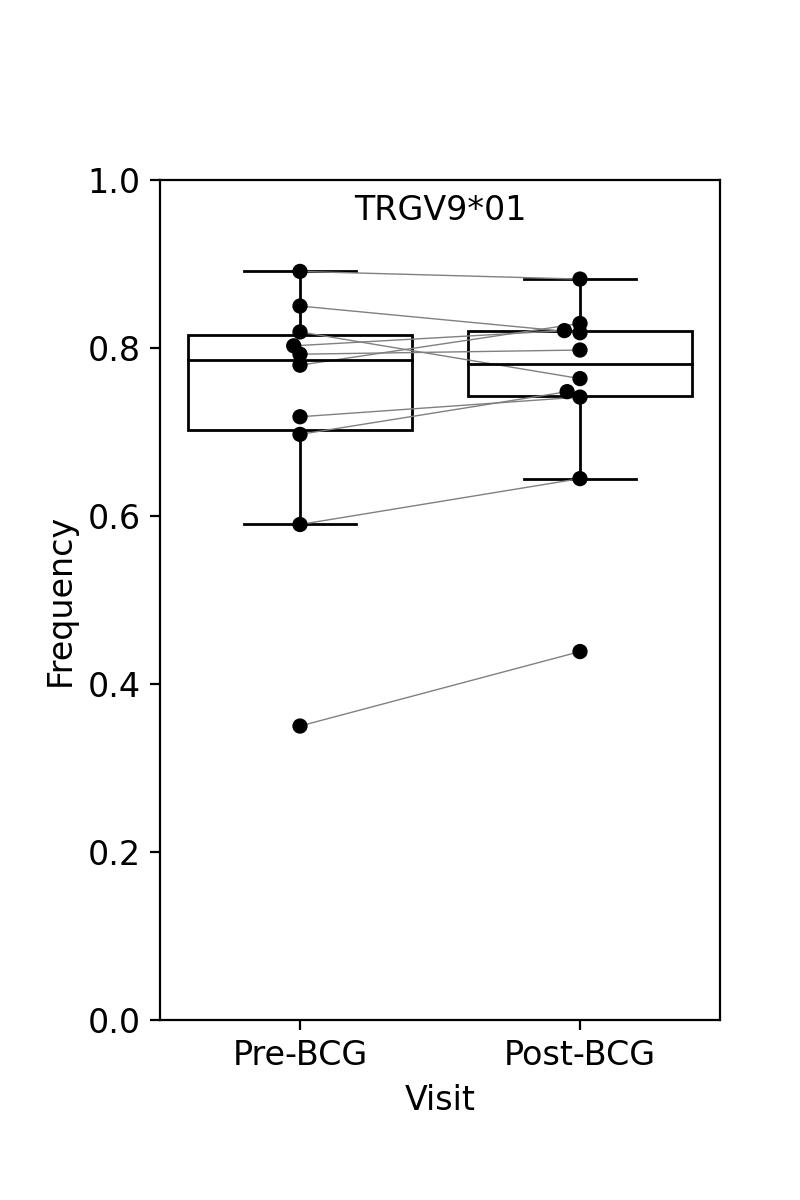 | B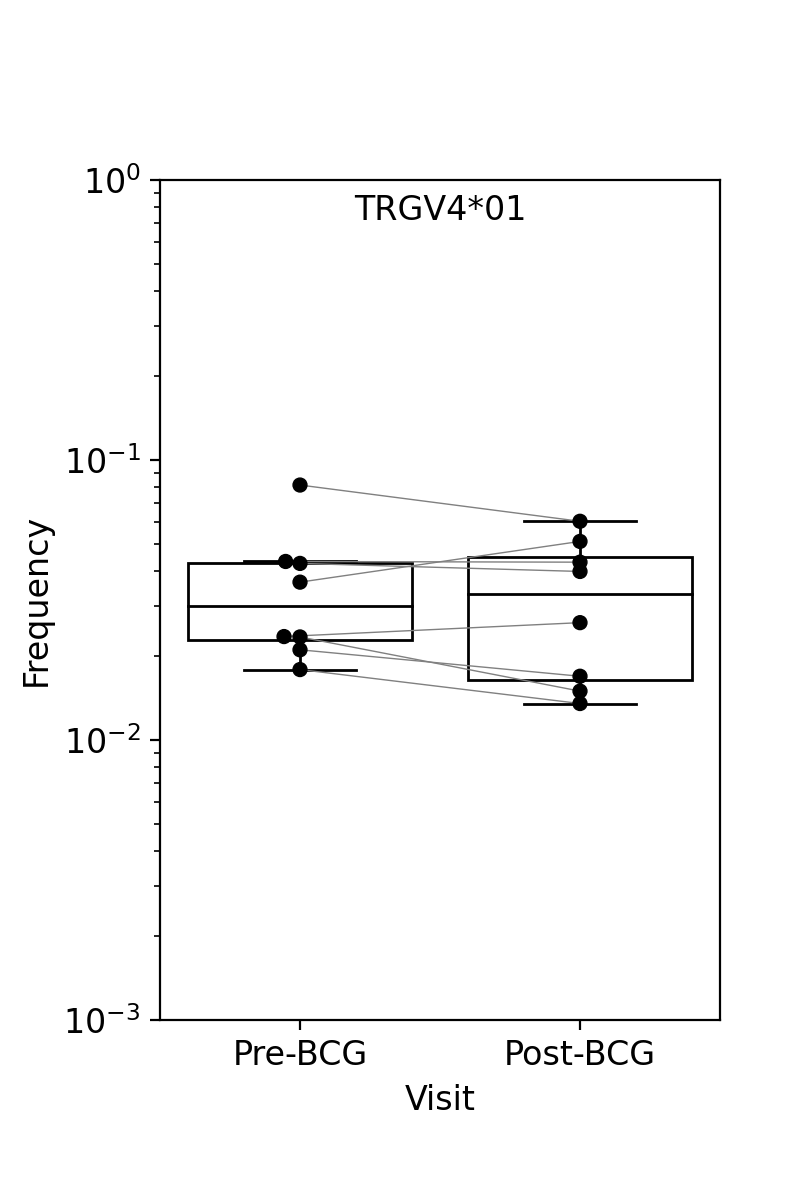 |
| --- | --- |
| C 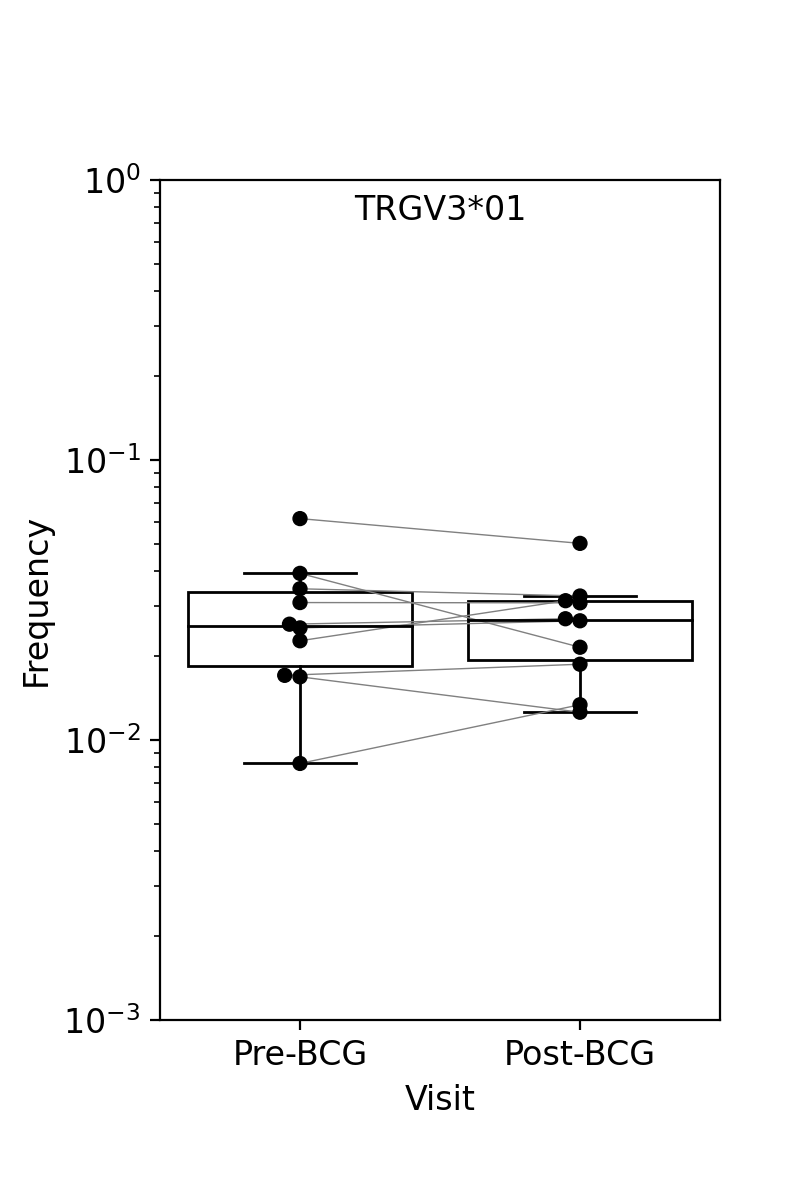 | D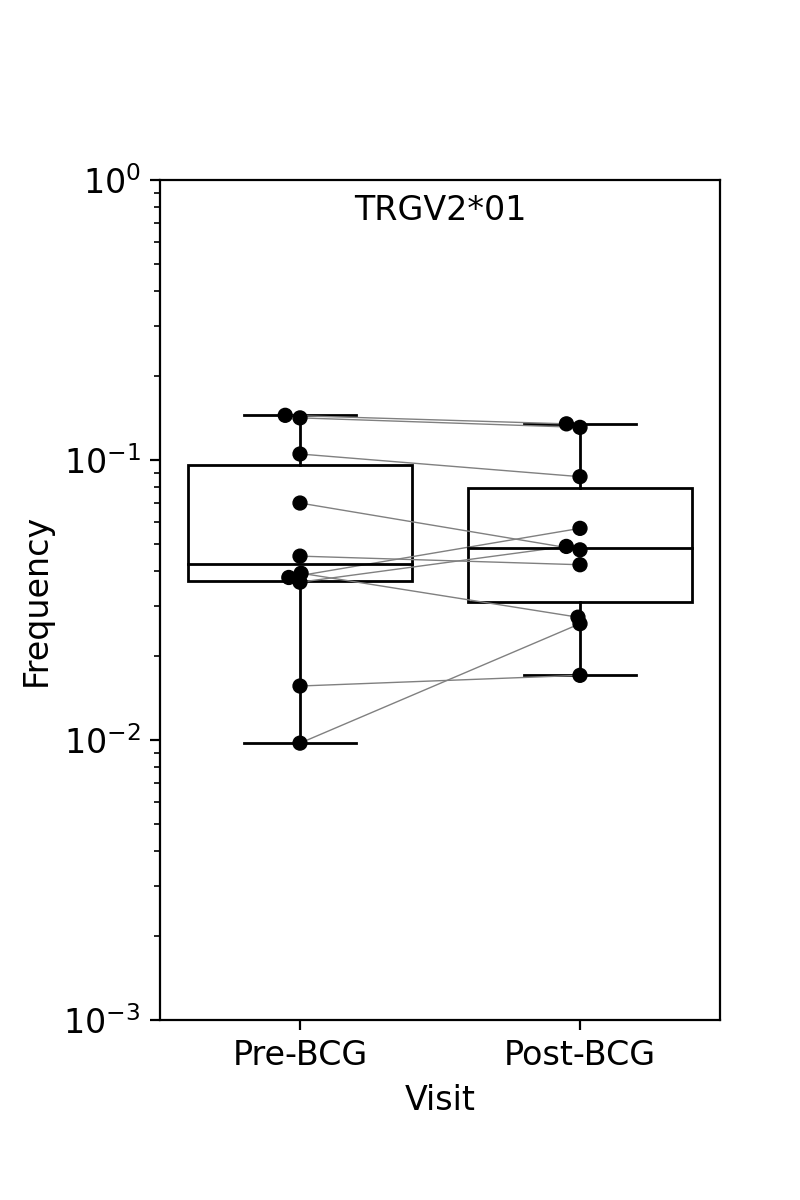 |

**Supplemental Figure S1.** **Relative abundance of TCRγ Variable region (V) genes among bulk sequenced T cells**. Frequency of the four most frequent TCRγ V-genes are shown before and after BCG vaccination; no significant differences were found (paired signed-rank test, FWER-p > 0.05 and FDR-q > 0.05).

| A 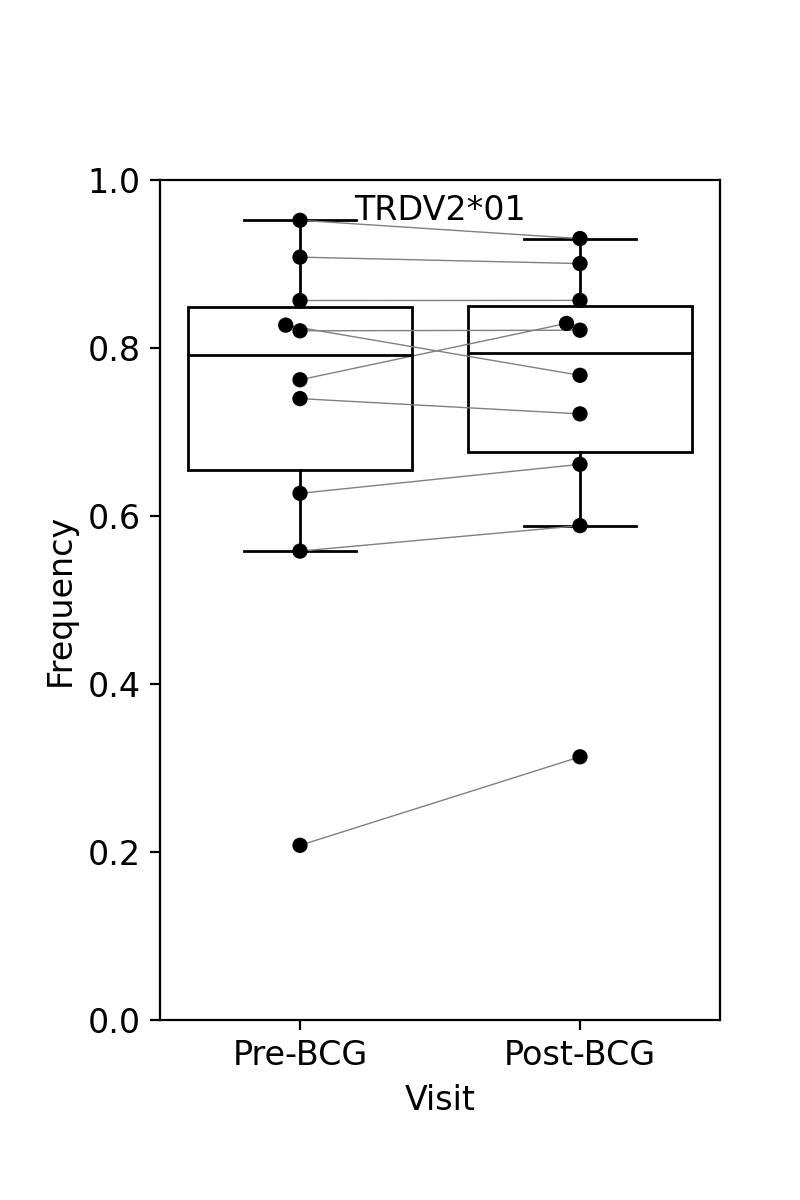 | B 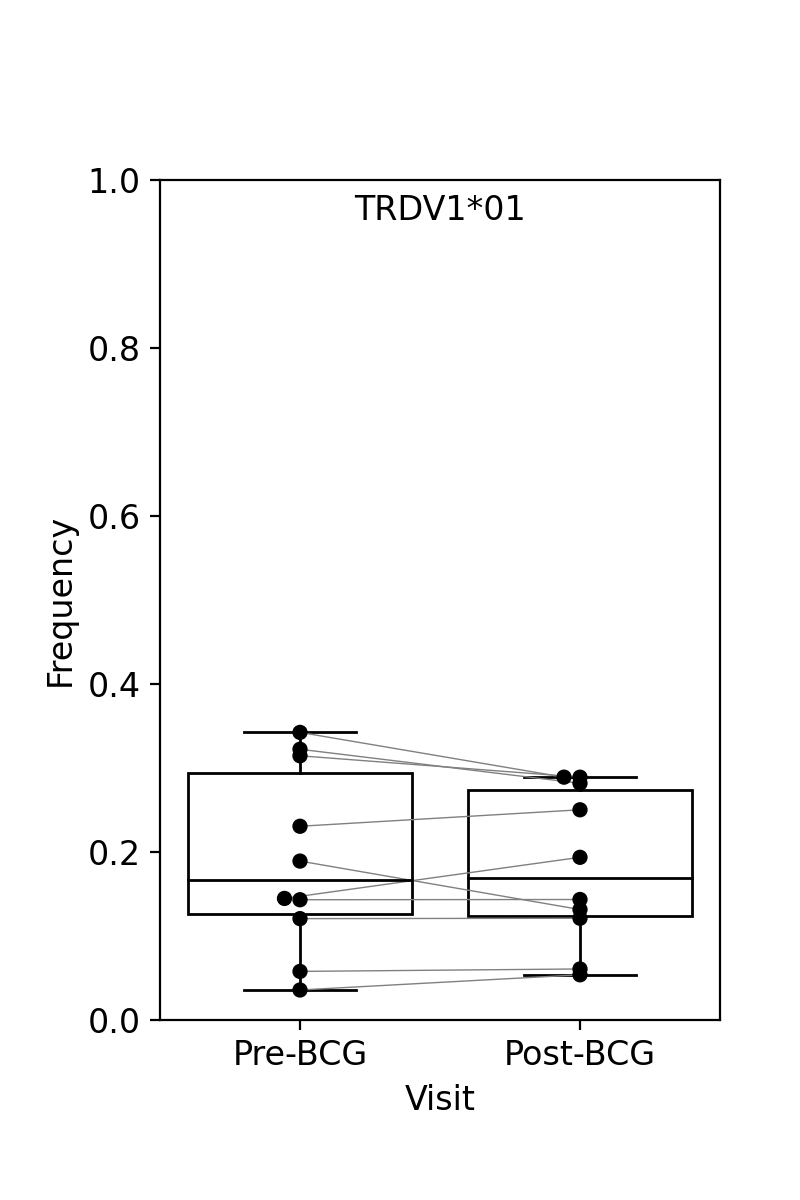 |
| --- | --- |
| C 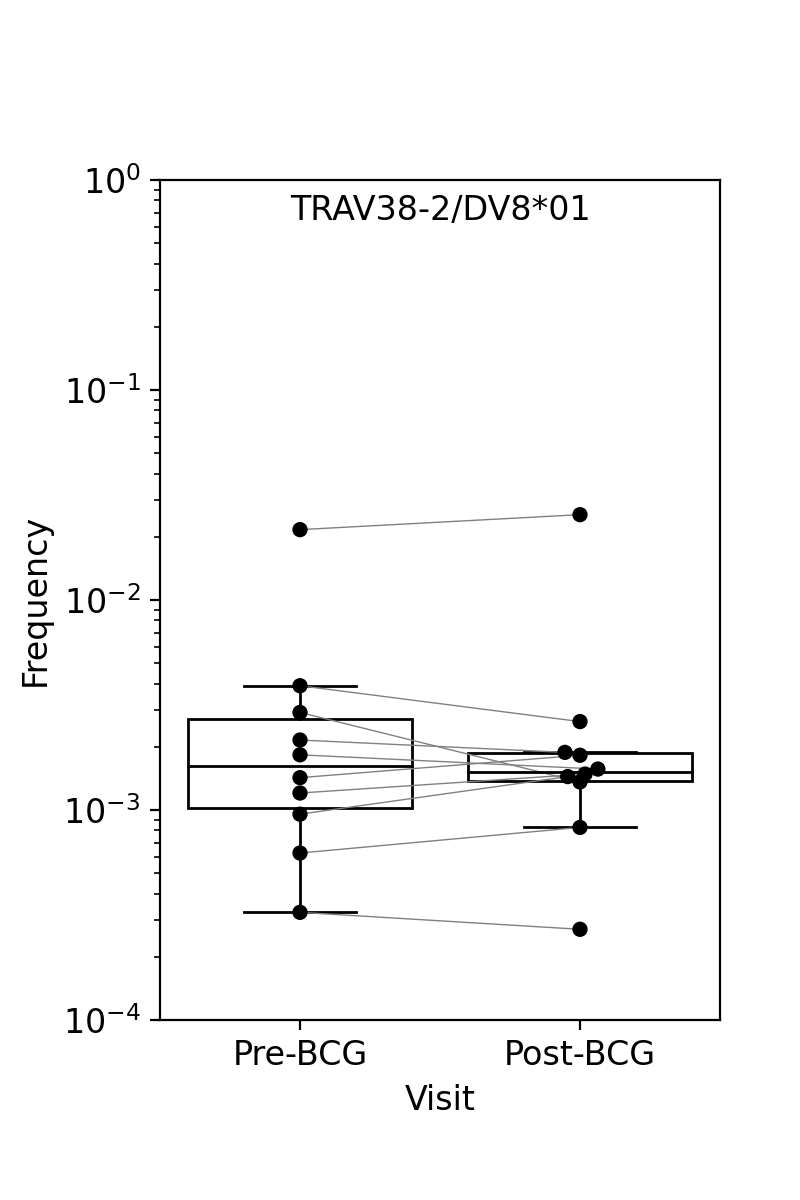 | D 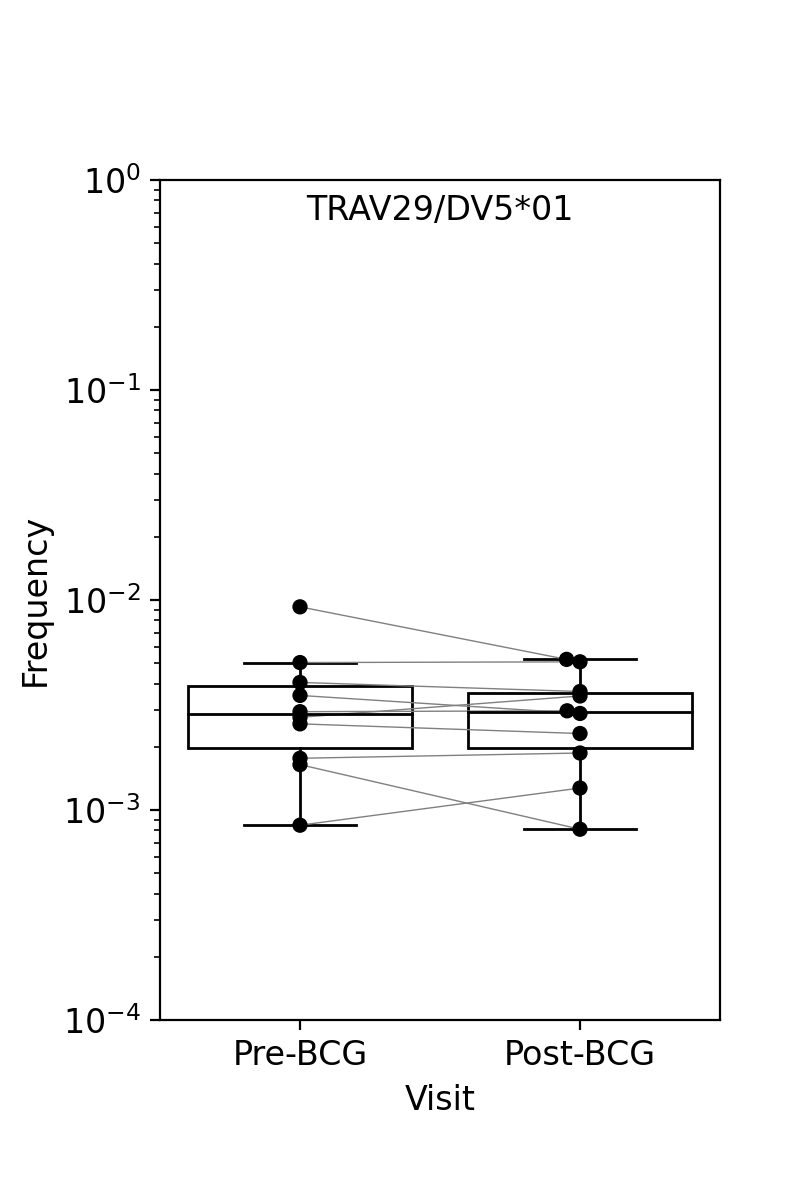 |

**Supplemental Figure S2.** **Relative abundance of TCRδ Variable region (V) genes among bulk sequenced T cells**. Frequency of the four most frequent TCRδ V-genes are shown before and after BCG vaccination; no significant differences were found (paired signed-rank test, FWER-p > 0.05 and FDR-q > 0.05).

| **A. TRGV9**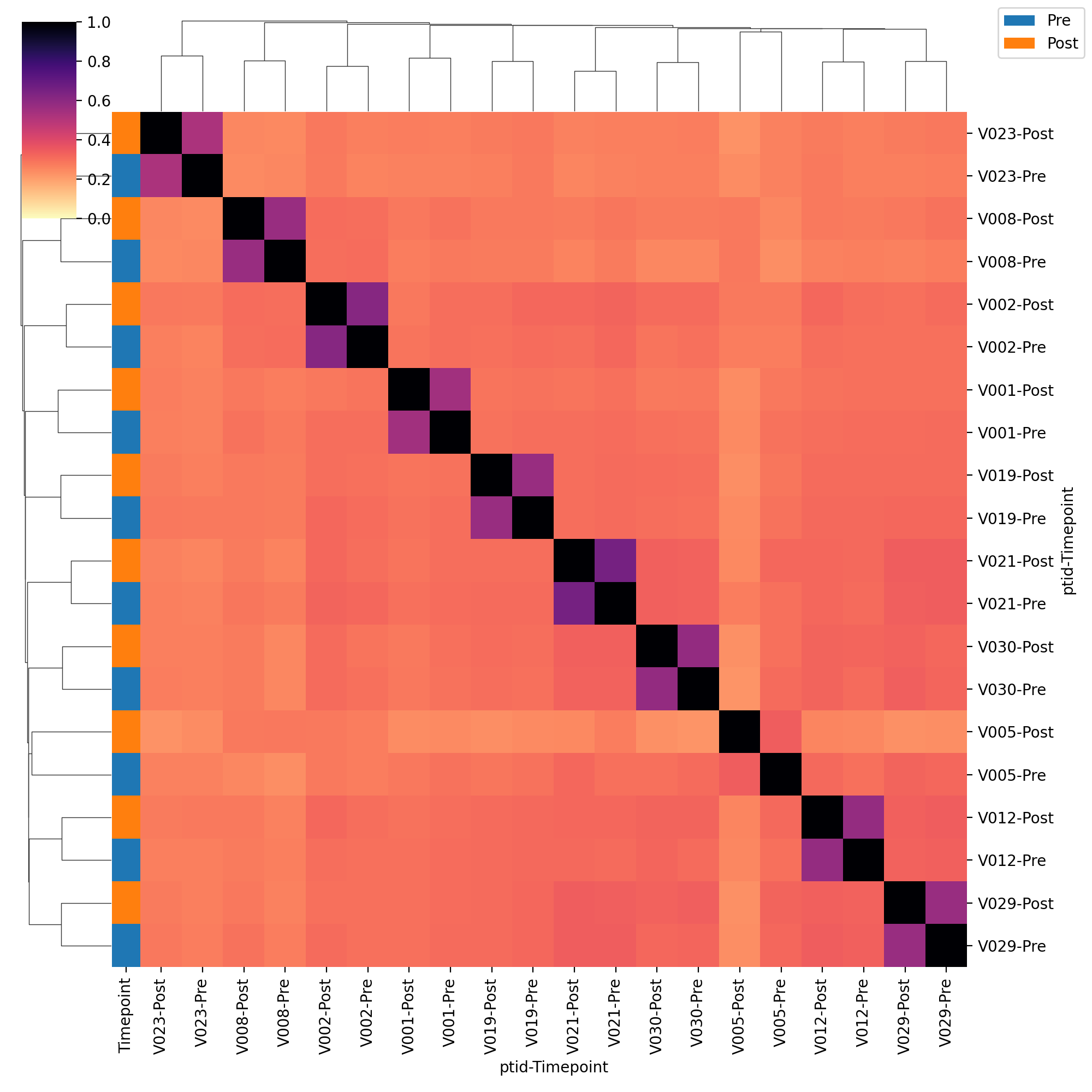 | **B. Non-TRGV9**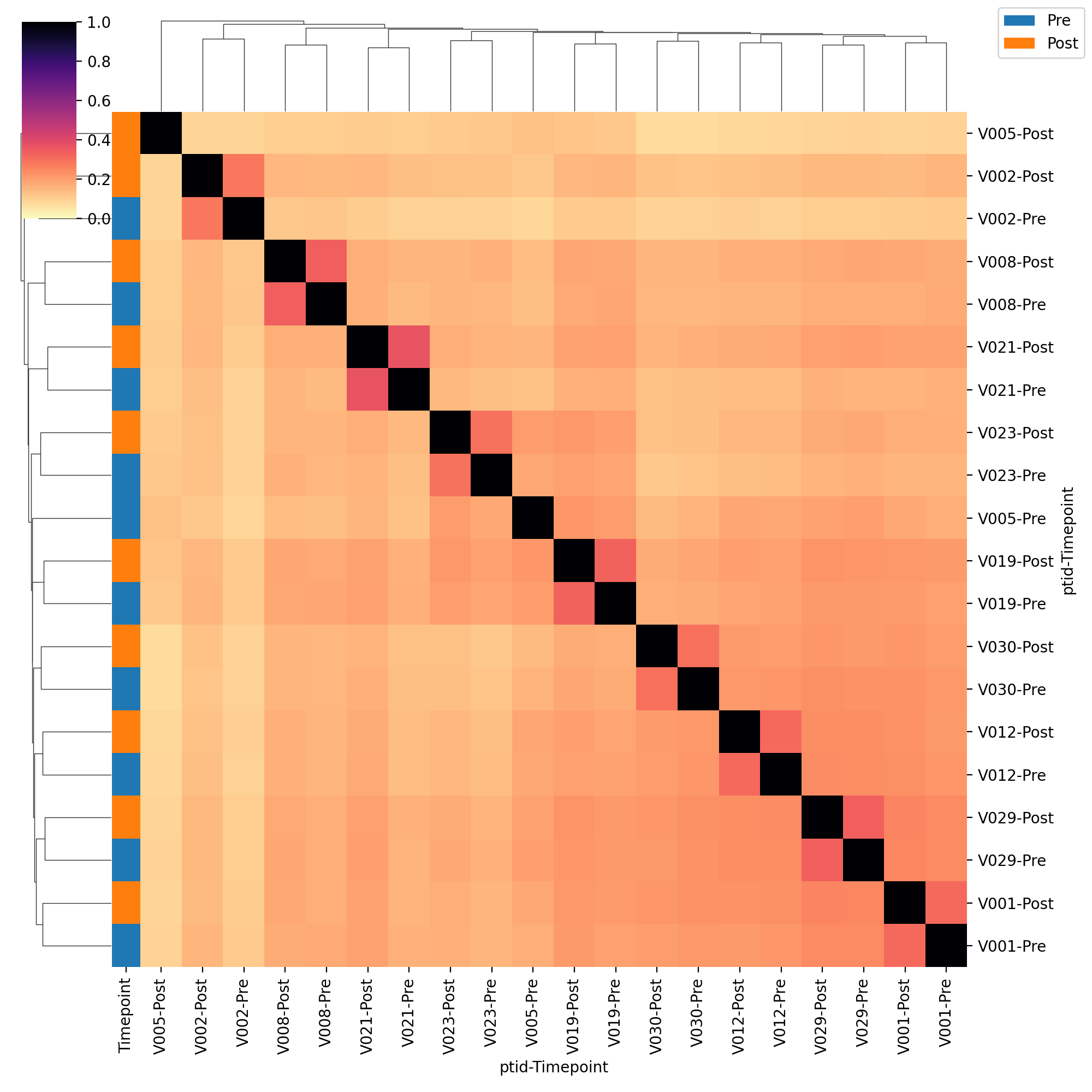 |
| --- | --- |
| **C. TRDV2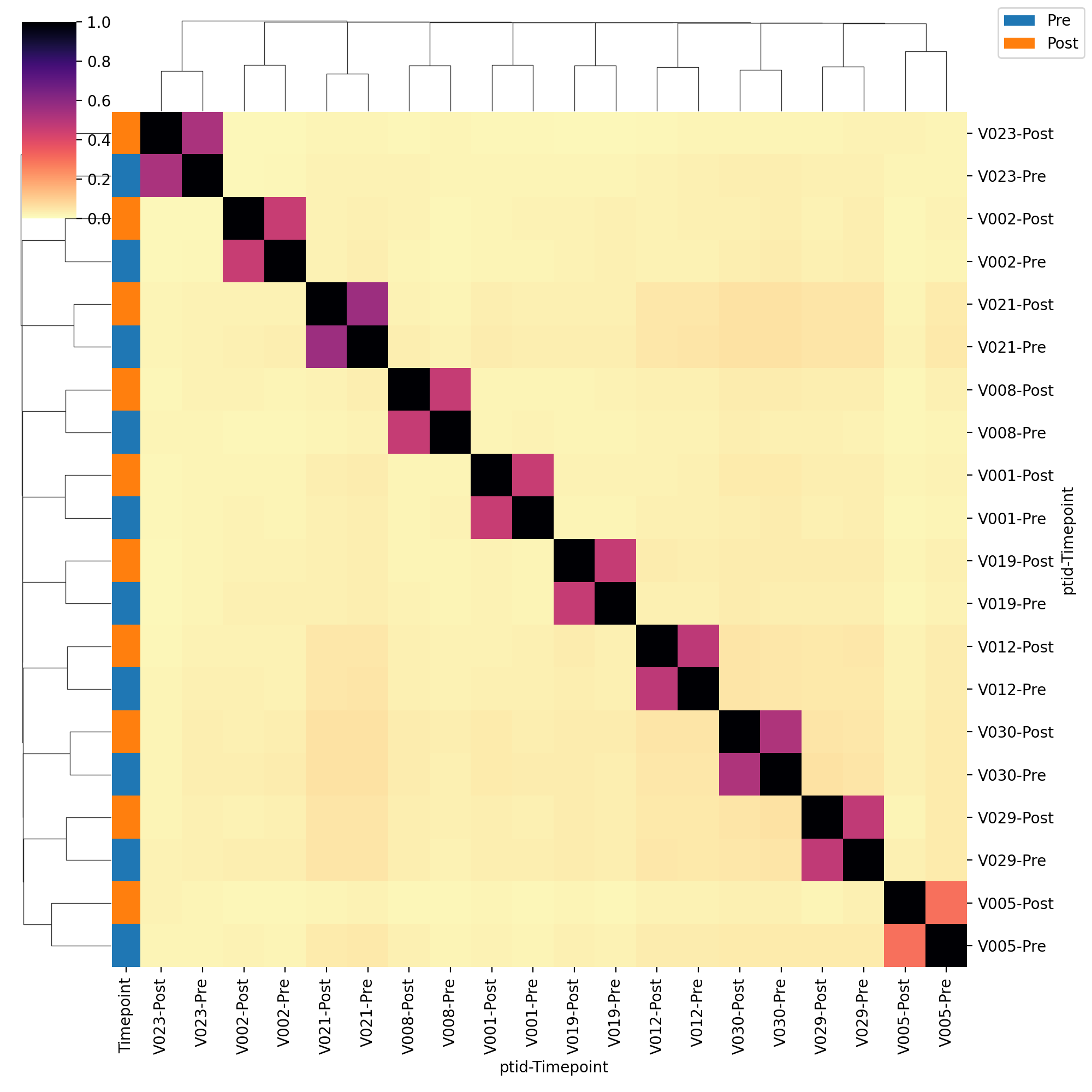** | **D. Non-TRDV2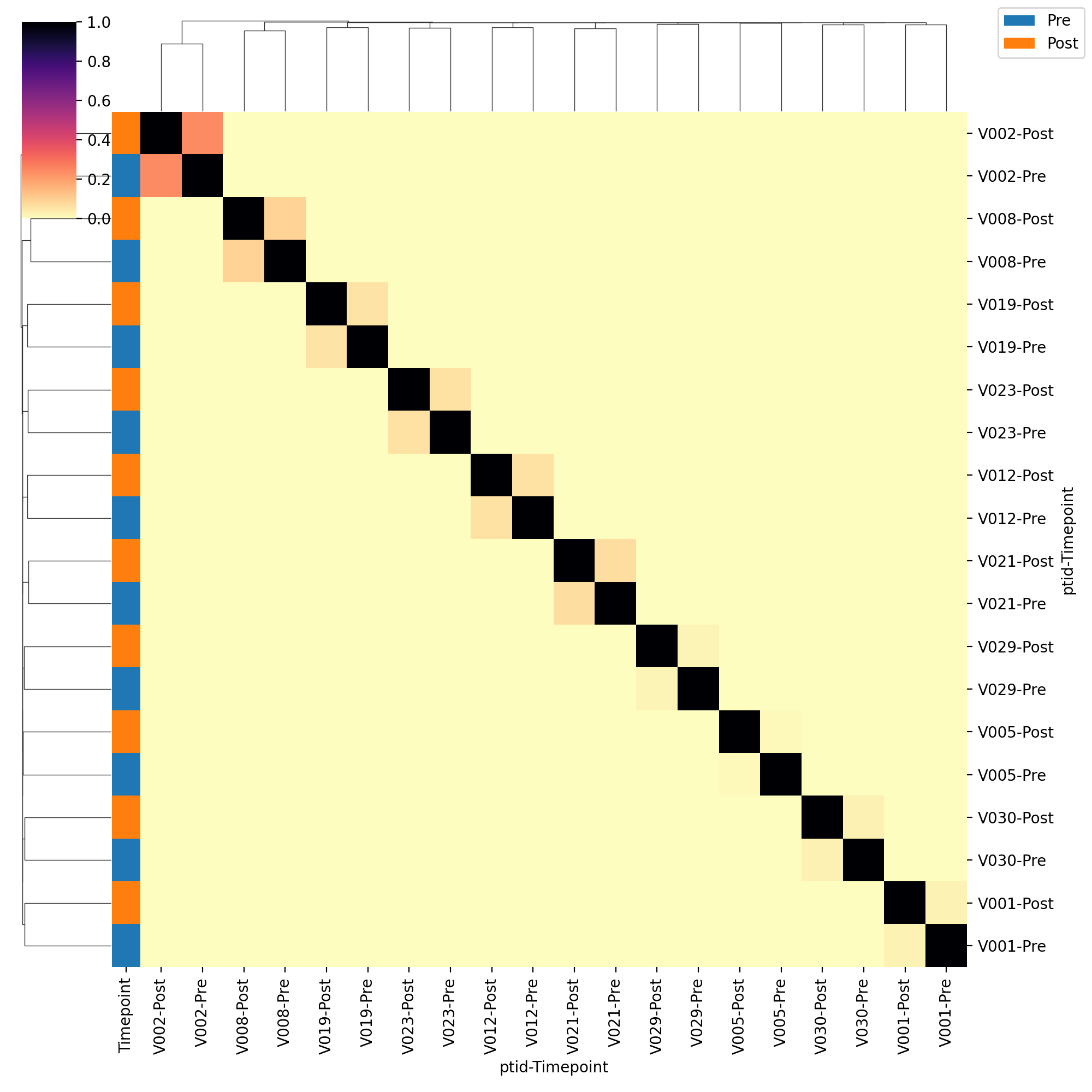** |

**Supplemental Figure S3. TCR single-chain repertoire sharing**. Heatmaps showing the (A) TRGV9*01^+^ , (B) TRGV9*01^-^ , (C) TRDV2*01^+^ , and (D) TRDV2*01^-^ clonotypes shared between pairs of samples. Sharing constitutes a single-chain clonotype that matches in the V-gene, J-gene and CDR3 amino acids (proportion shared = 2*N_shared_ / [N_A_ + N_B_]). Samples are labeled according to the volunteer number (VX) and the timepoint relative to BCG vaccination. Sample timepoint is also indicated by the color bar.

| **A. TCRγ**  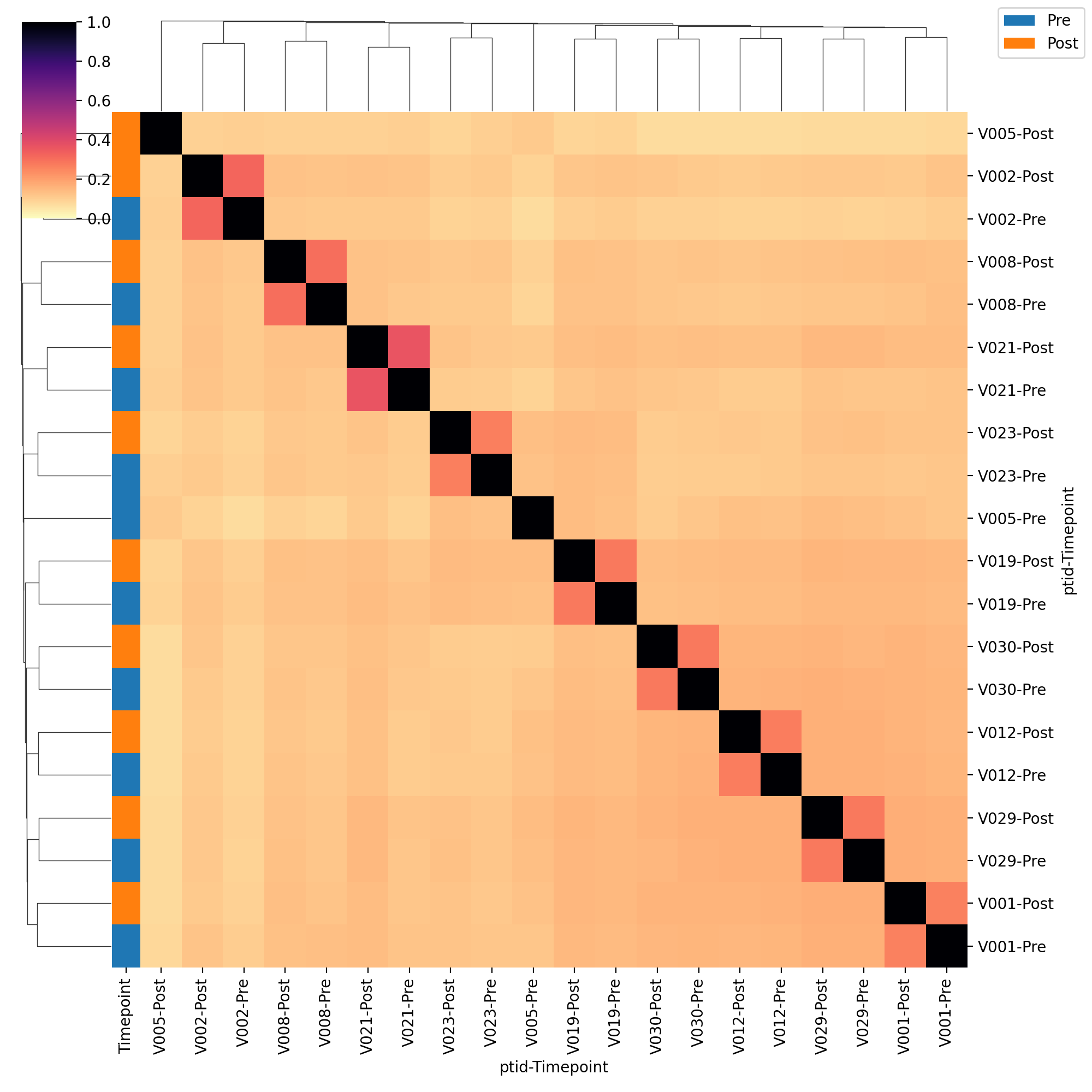 | **B. TCRδ**  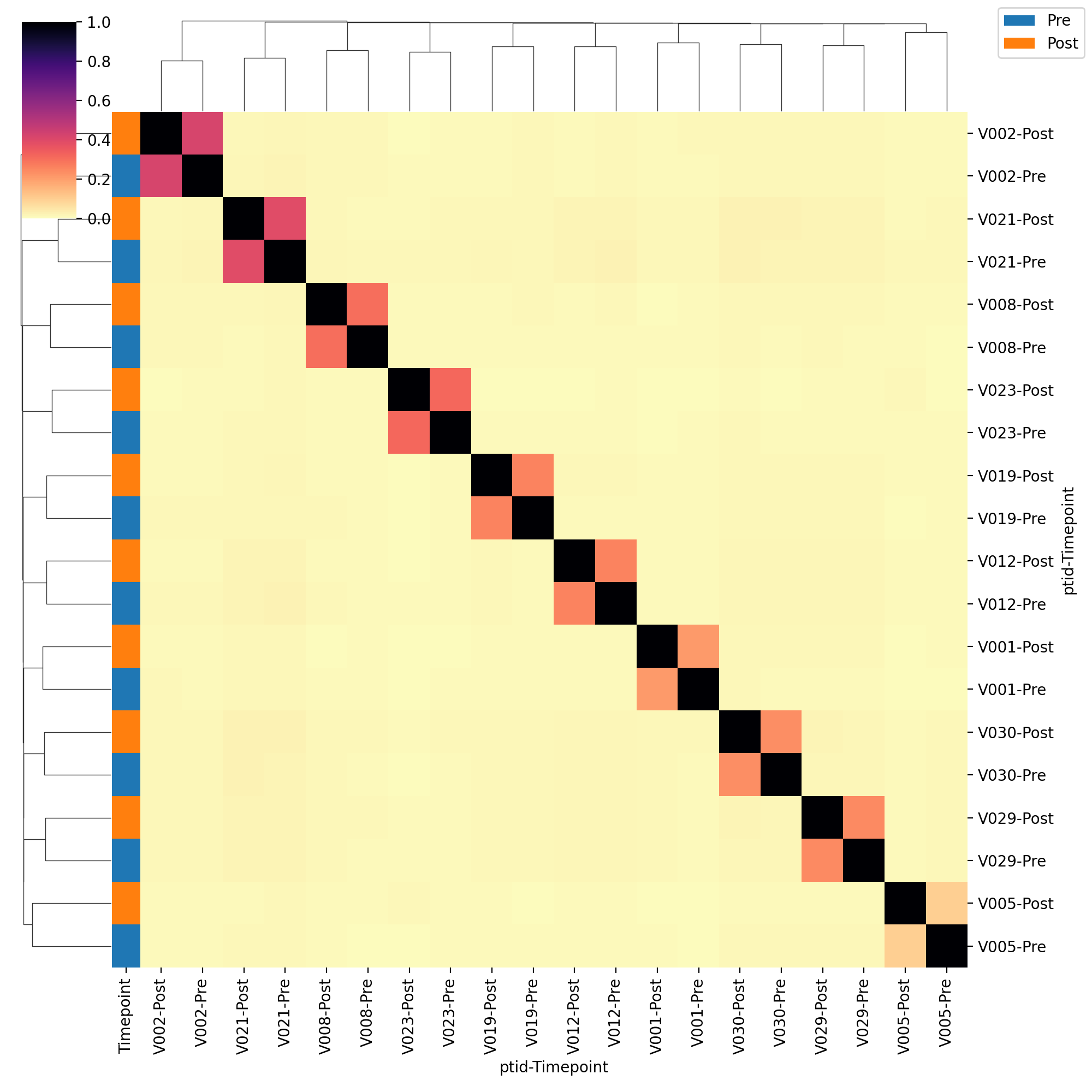 | **C. TCRβ**  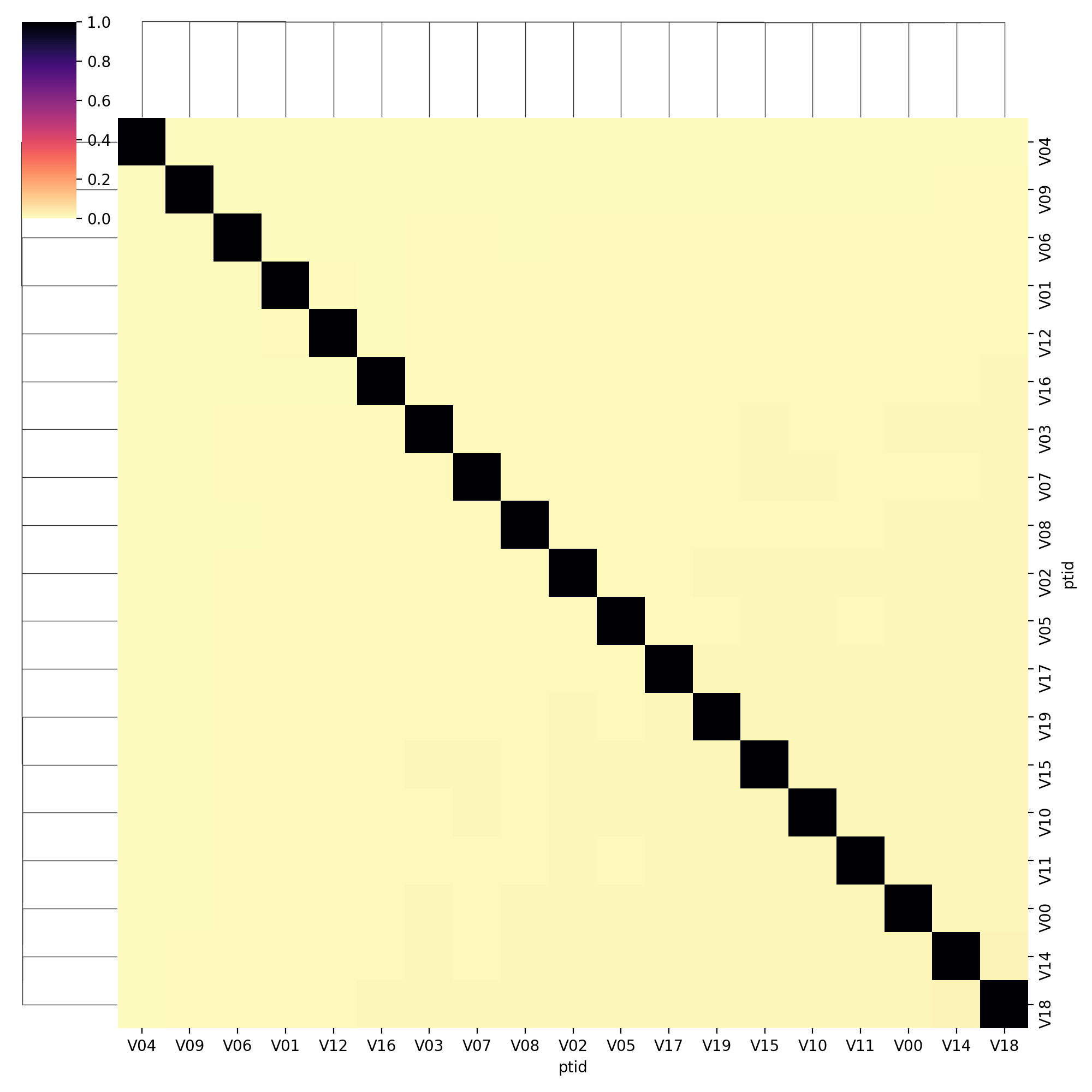 |
| --- | --- | --- |

**Supplemental Figure S4. TCR repertoire sharing among study participants and healthy controls**. Heatmap showing the proportion of (A) TCRγ (B) TCRδ and (C) TCRβ chain sequences from one sample that match (V-gene, J-gene and CDR3 amino-acids) a chain from another sample (proportion shared = 2*N_shared_ / [N_A_ + N_B_]). TCRγ and TCRδ data combine chains using all V-genes from study participants. TCRβ repertoires obtained from healthy control volunteers at a single time point (n = 20; Emerson et al., 2017)

| A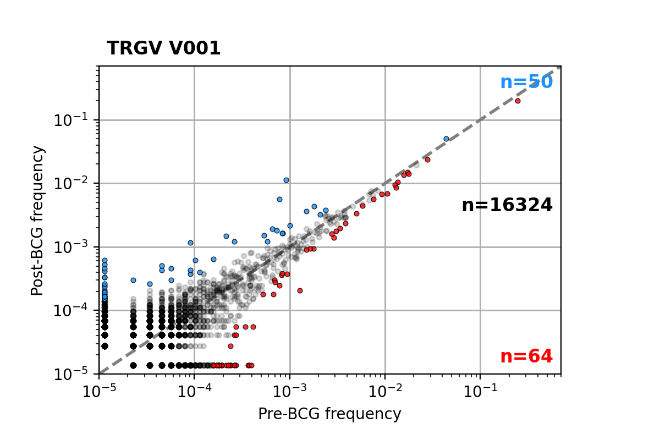 | B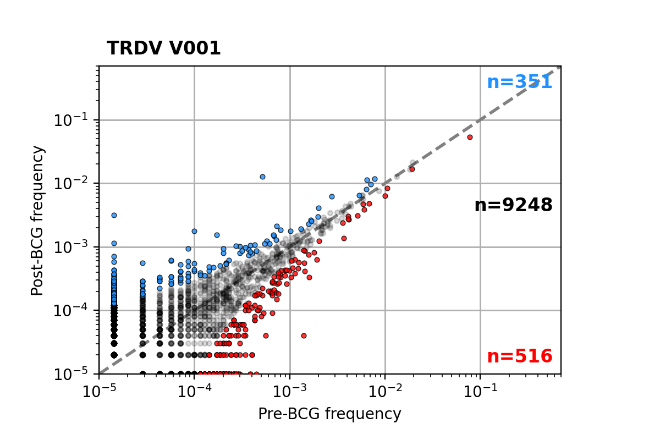 |
| --- | --- |
| C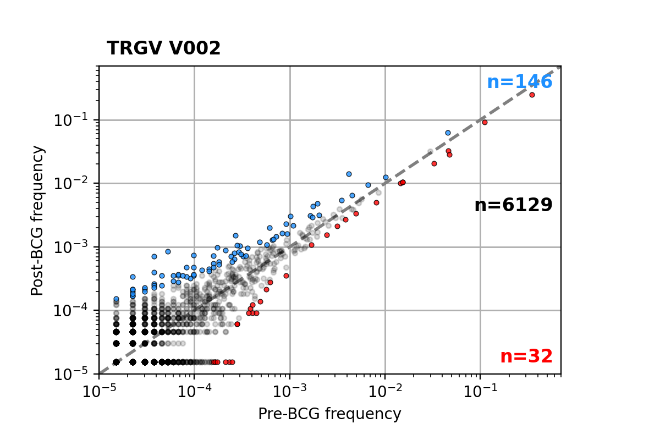 | D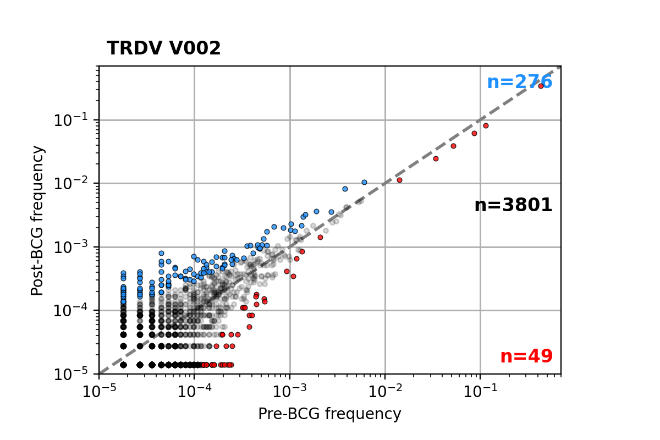 |
| E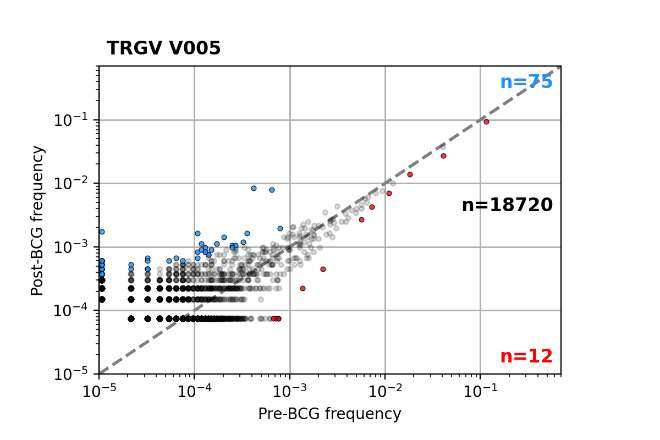 | F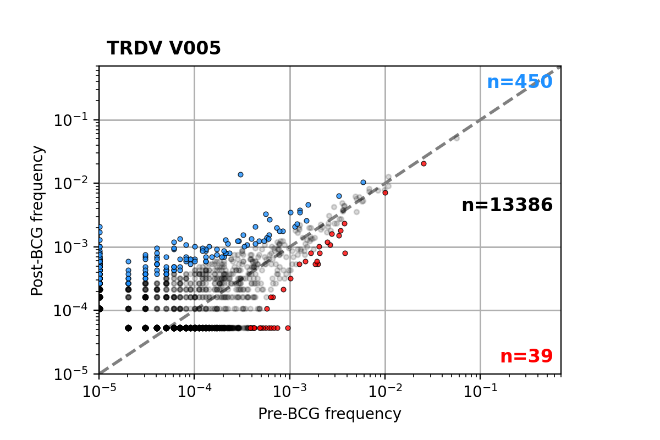 |
| G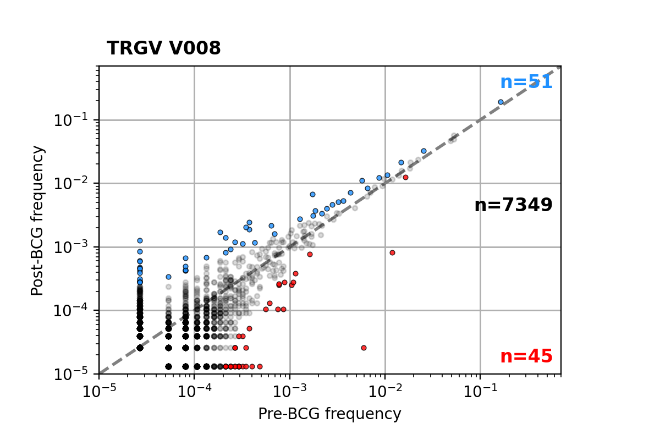 | H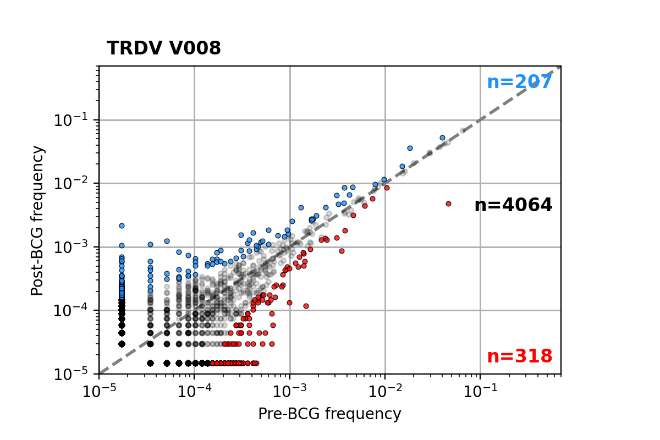 |
| I 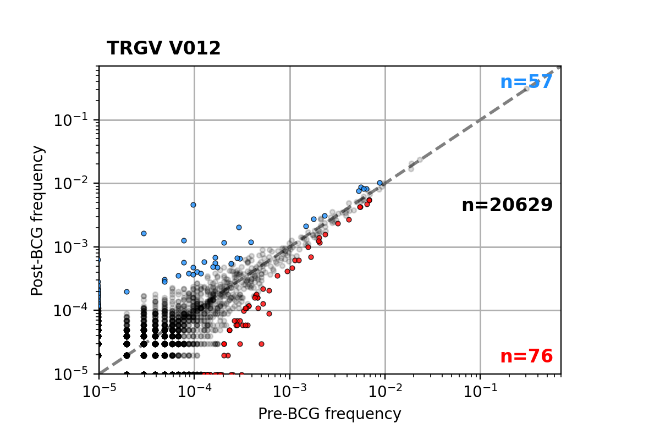 | J 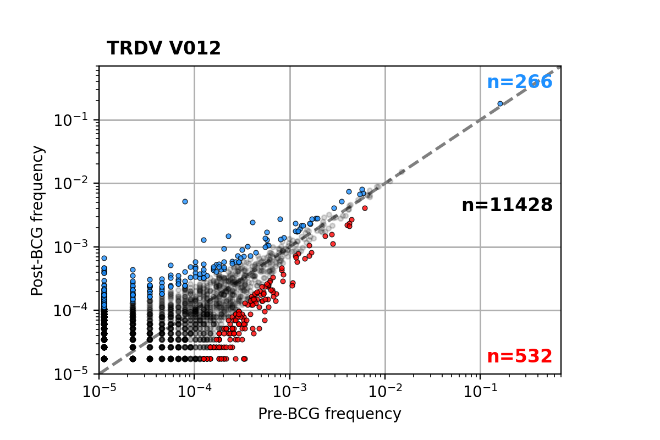 |
| K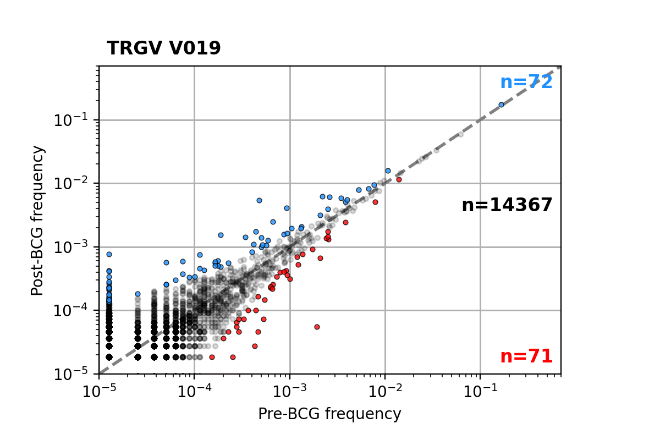 | L 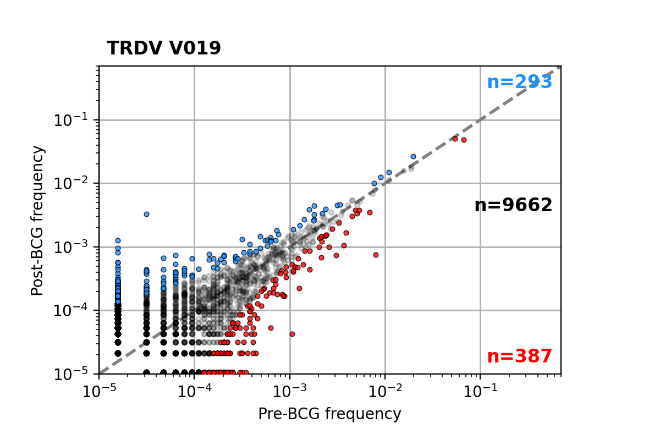 |
| M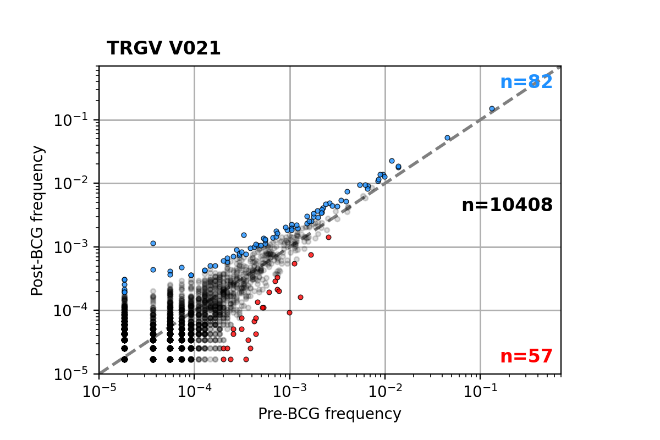 | N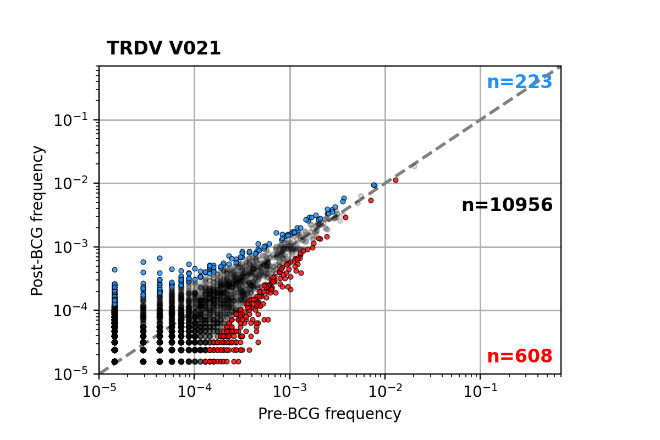 |
| O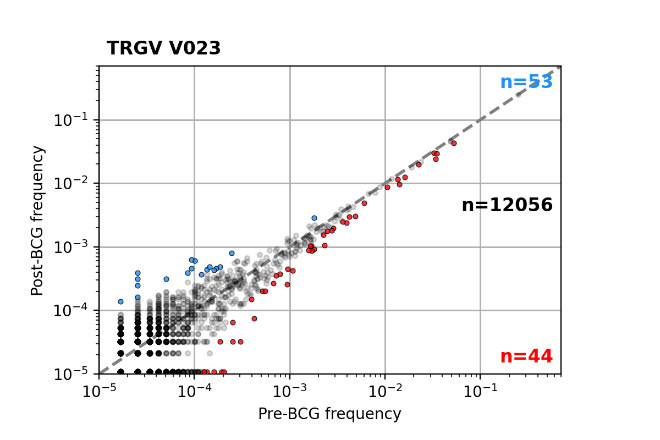 | P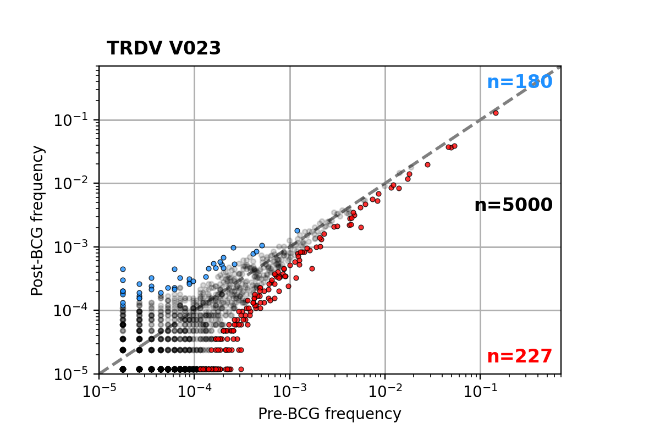 |
| Q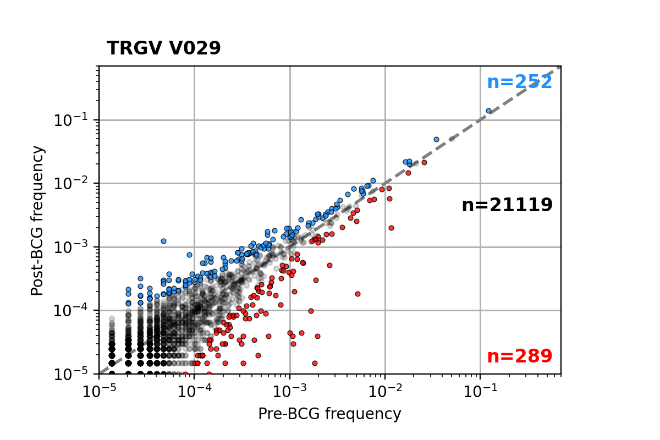 | R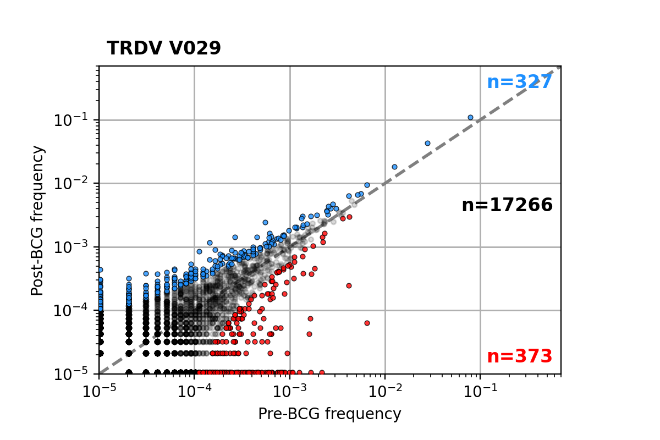 |
| S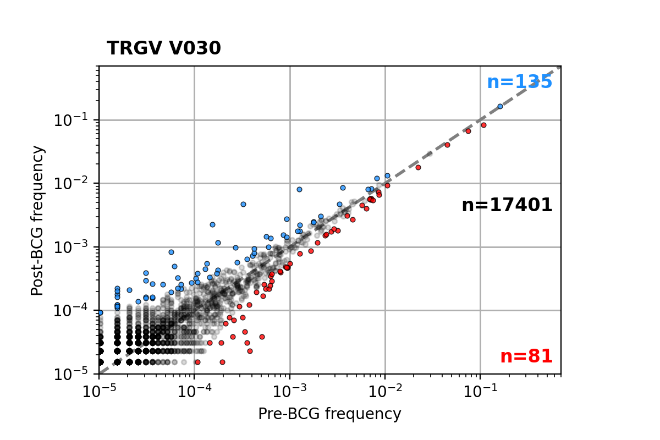 | T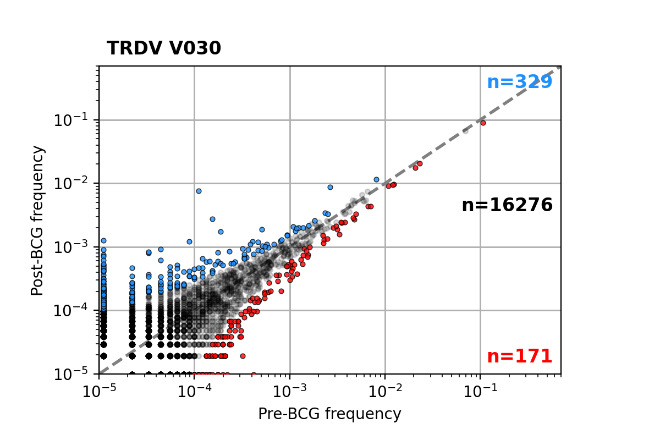 |

**Supplemental Figure S5.** **Frequency scatter plots of per participant clonotype analysis.** Pre- and Post-BCG TCRγ and TCRδ repertoires were analyzed from each participant (A – T; n = 10 participants). Each symbol represents one TCR single-chain clonotype plotted as pre vs. post BCG frequency. Symbols colored blue (red) show clonotypes with a FDR-adjusted q-value < 0.05 indicating a significant increase (decrease) in frequency after BCG.

| **A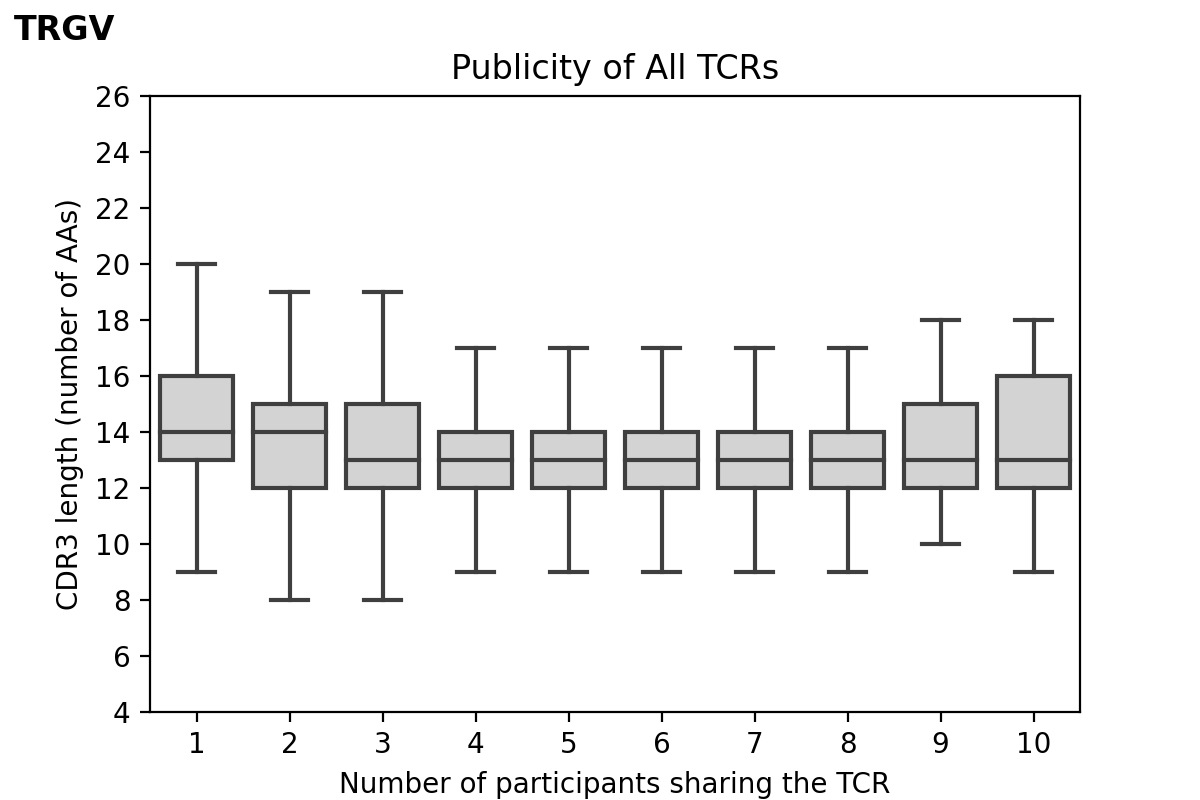** | **C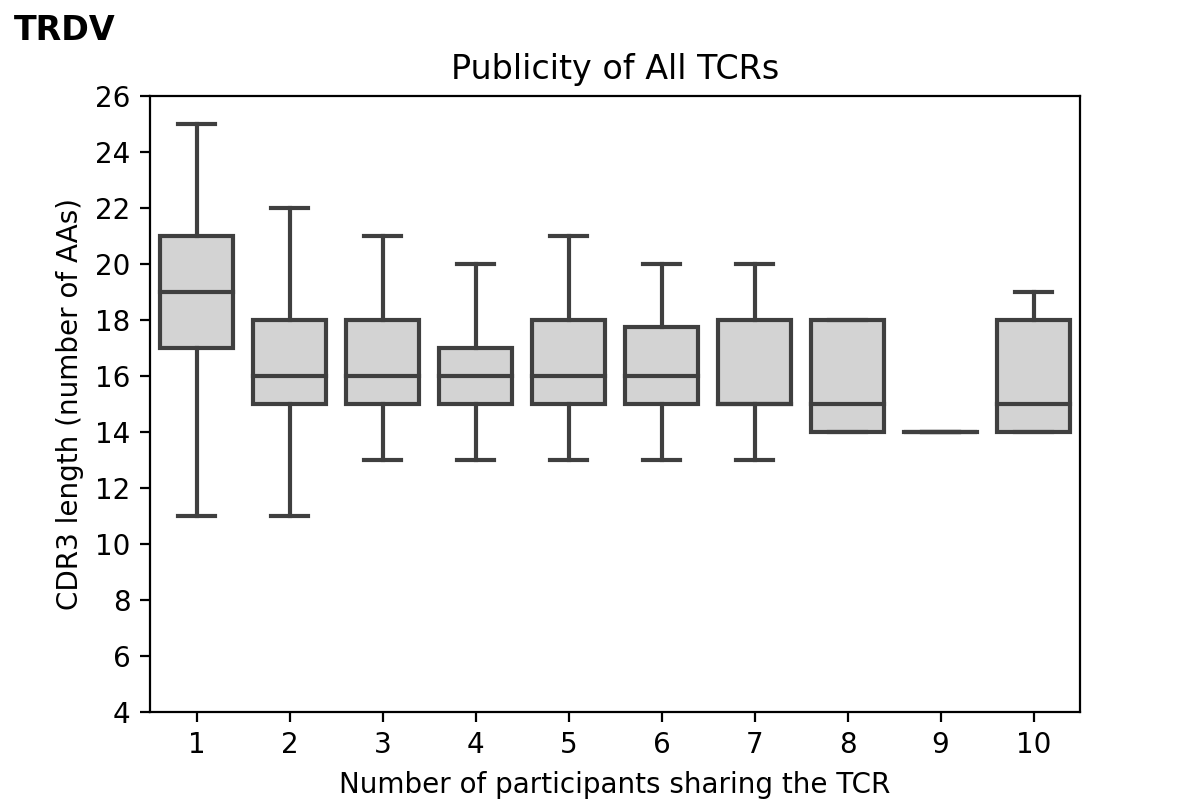** |
| --- | --- |
| **B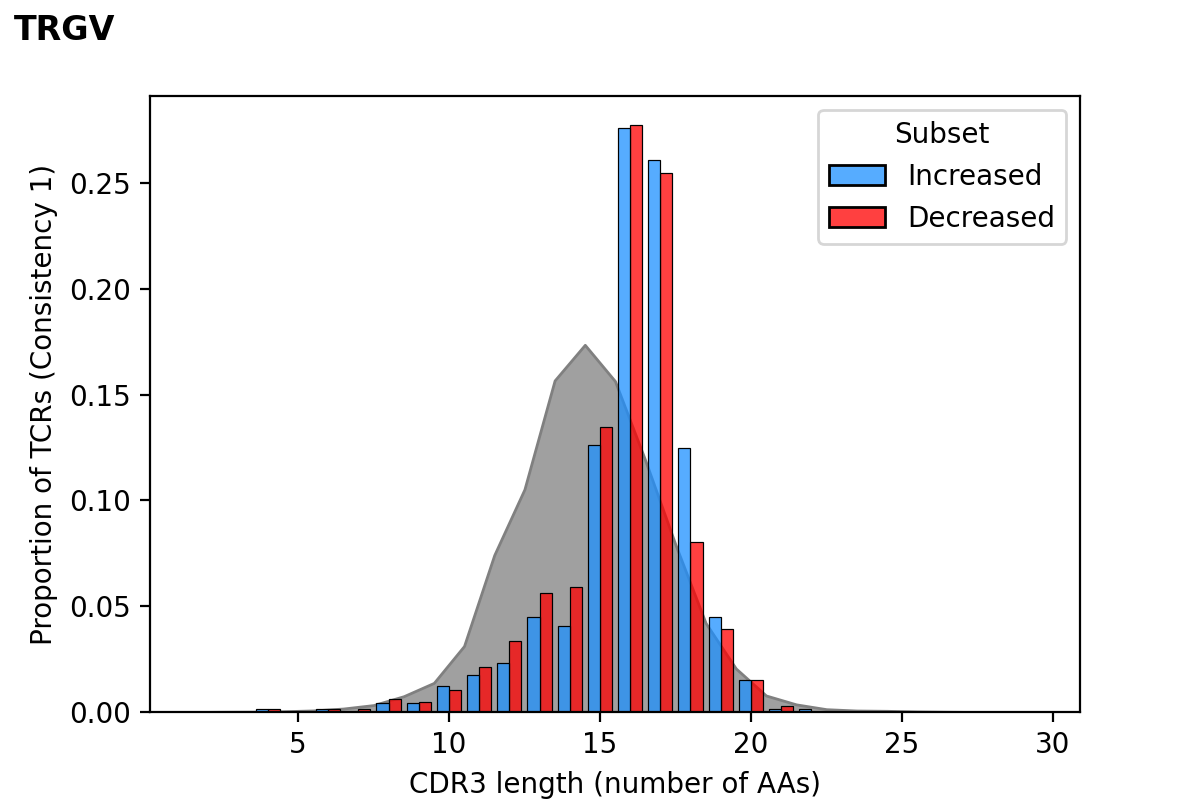** | **D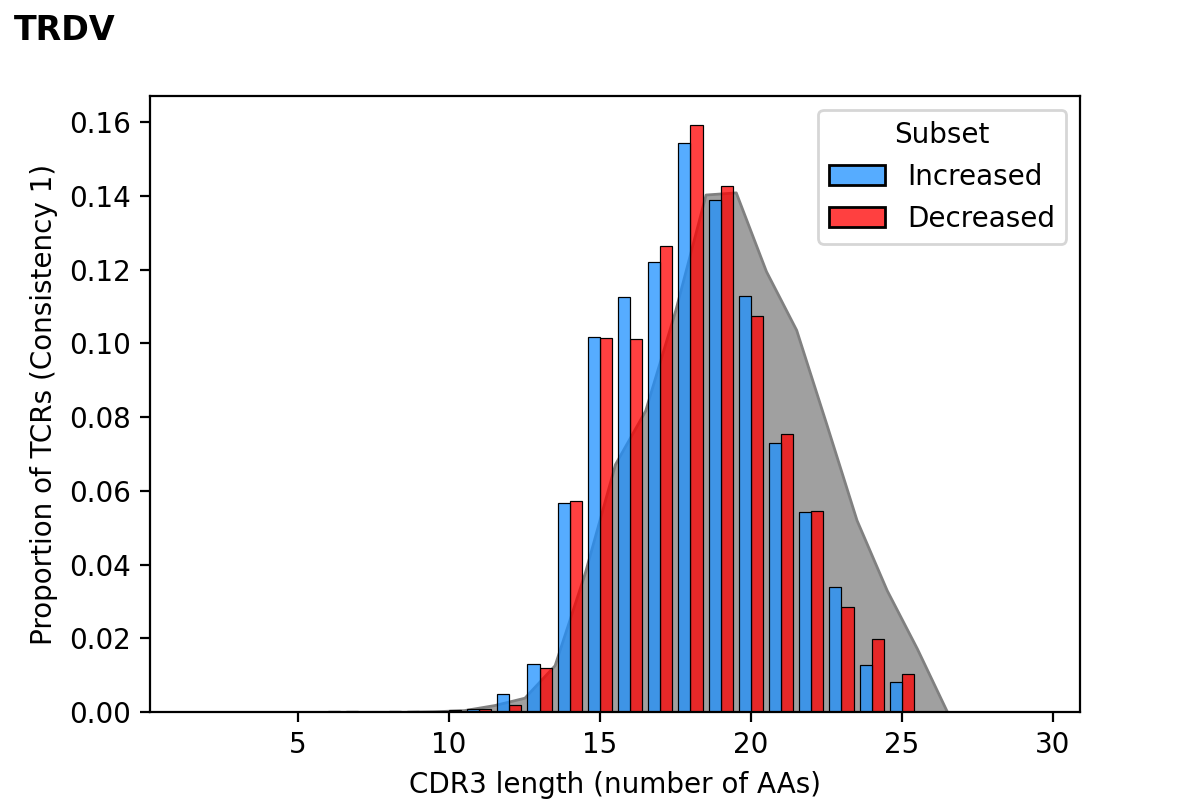** |

**Supplemental Figure S6. TCRγ TCRδ CDR3 length distributions.** Sharing number of each unique TCRγ clonotype was computed as the number of participants (out of 10) that had at least one sample containing the TCR sequence (matched V/J-gene and CDR3 amino-acids). (A) Boxplots indicate the median and interquartile range (IQR) of the CDR3 length for TCRs categorized by their sharing number; whiskers indicate the extent of 1.5 times the IQR or the most extreme length. (B) A frequency histogram of CDR3 length was created from all unique TCRγ clonotypes (gray region) or only the TCRs that were significantly increased or decreased in ≥1 individual. (C, D) Parallel analyses were conducted for the TCRδ repertoires.

| 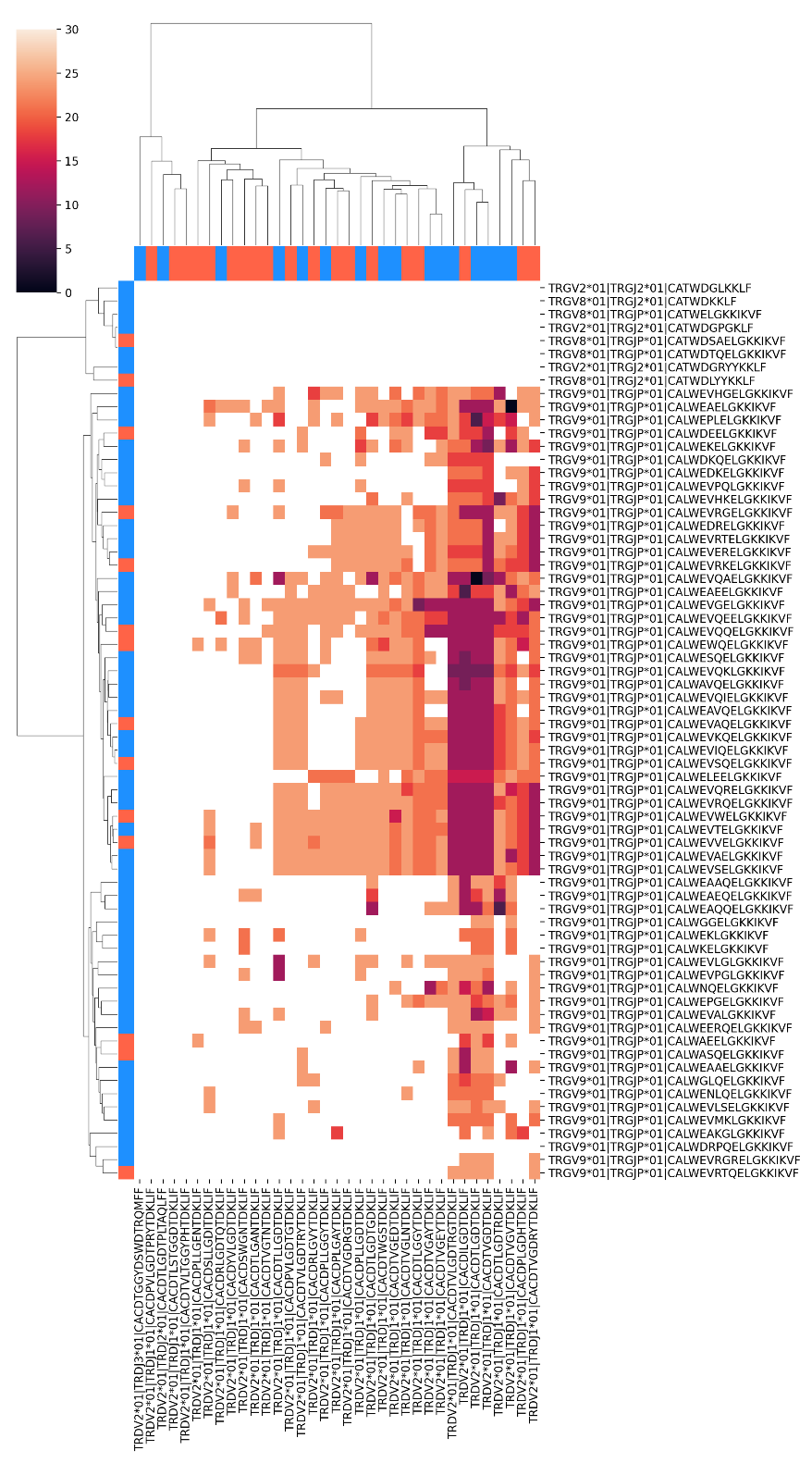 | **Supplemental Figure S7. Pairings of** **TCRγ and TCRδ chains in single-cell data.** TCRγ and TCRδ single-chains that were consistently increased or decreased in abundance after BCG (consistency ≥2) were subject to all combinations of pairings as candidate paired-chain γδTCRs. A dataset of paired-chain single-cells was sequenced from PBMC sampled before and after BCG vaccination in two volunteers. Each candidate pair was scored based on the TCRdist distance to the closest observed pairing (12 TCRdist units is approximately 1 aa substitution in the CDR3 of either chain). Black squares (TCRdist = 0) indicate that the exact γδTCR sequence was observed in at least one cell in the dataset (there were 3 such pairings). White squares indicate TCRs that were not similar to any observed γδTCR, and colors on the color scale indicate the TCRdist distance to the most similar single-cell γδTCR. The color bar along the top and left side of the heatmap show whether the single-chain TCR was consistently increased (blue) or consistently decreased (red) after BCG. |
| --- | --- |

**
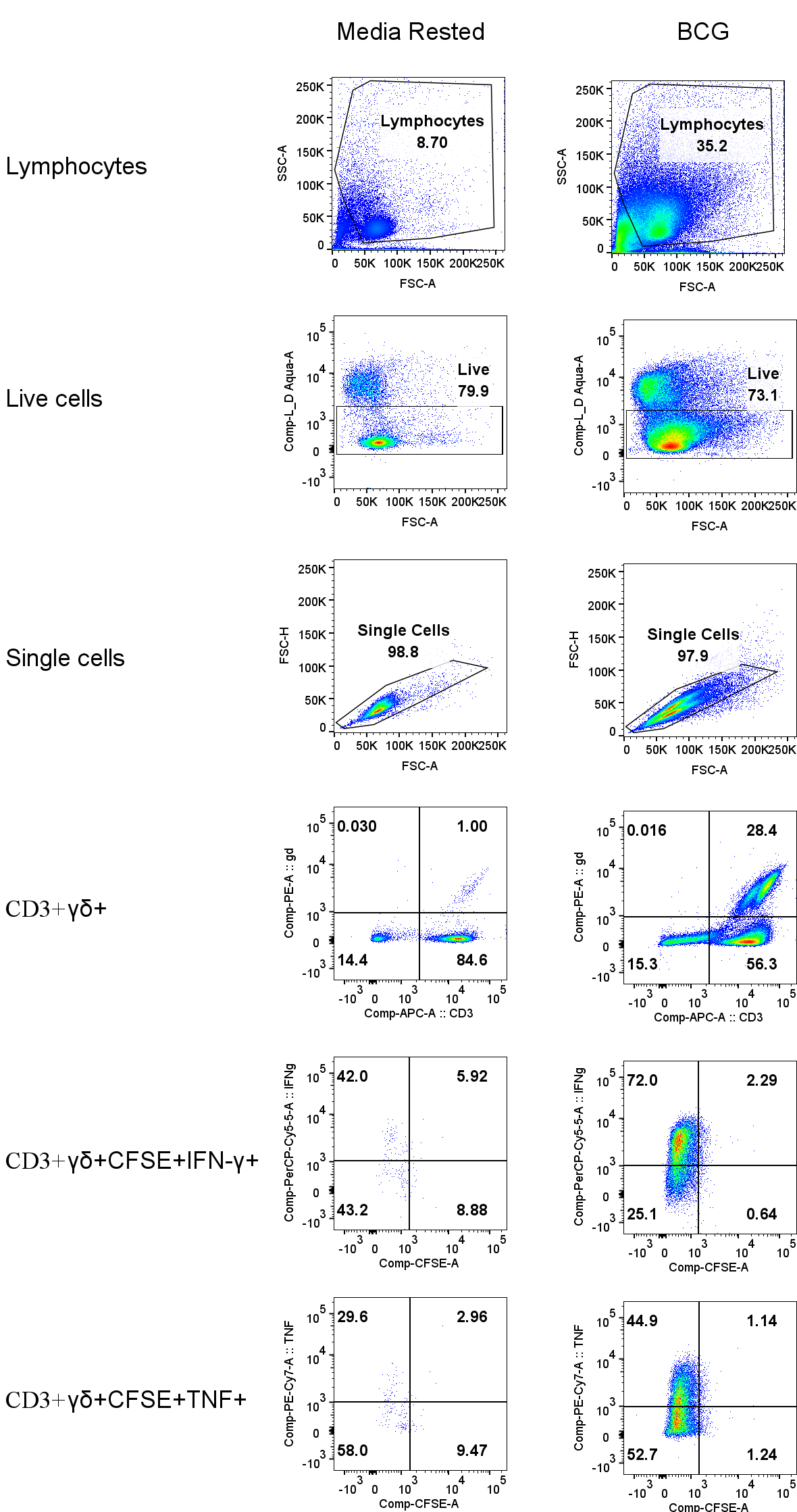
**

**Supplemental Figure S8. Representative FACS plots.**

Representative gating strategy employed for the identification of γδ cells from lymphocytes using multi-parametric flow cytometry. Side scatter area (SSC-A) vs forward scatter area (FSC-A) was used to identify lymphocytes followed by exclusion of non-viable cells by use of viability dye. Then we excluded doublets by forward scatter height (FSC-H) vs FSC-A, γδ T cells were identified using anti-CD3 and anti-γδ monoclonal antibodies, whilst CFSE^LO^ cells were identified cells that had proliferated cells. Cytokine producing cells were identified by using anti-IFN-γ and anti-TNF antibody.
